# Cation Homeostasis: Coordinate Regulation of Polyamine and Magnesium Levels in Salmonella

**DOI:** 10.1128/mbio.02698-22

**Published:** 2022-12-07

**Authors:** Yumi Iwadate, Yekaterina A. Golubeva, James M. Slauch

**Affiliations:** a Department of Microbiology, University of Illinois at Urbana-Champaign, Urbana, Illinois, USA; University of Utah

**Keywords:** polyamines, magnesium, *Salmonella*

## Abstract

Polyamines are organic cations that are important in all domains of life. Here, we show that in Salmonella, polyamine levels and Mg^2+^ levels are coordinately regulated and that this regulation is critical for viability under both low and high concentrations of polyamines. Upon Mg^2+^ starvation, polyamine synthesis is induced, as is the production of the high-affinity Mg^2+^ transporters MgtA and MgtB. Either polyamine synthesis or Mg^2+^ transport is required to maintain viability. Mutants lacking the polyamine exporter PaeA, the expression of which is induced by PhoPQ in response to low Mg^2+^, lose viability in the stationary phase. This lethality is suppressed by blocking either polyamine synthesis or Mg^2+^ transport, suggesting that once Mg^2+^ levels are reestablished, the excess polyamines must be excreted. Thus, it is the relative levels of both Mg^2+^ and polyamines that are regulated to maintain viability. Indeed, sensitivity to high concentrations of polyamines is proportional to the Mg^2+^ levels in the medium. These results are recapitulated during infection. Polyamine synthesis mutants are attenuated in a mouse model of systemic infection, as are strains lacking the MgtB Mg^2+^ transporter. The loss of MgtB in the synthesis mutant background confers a synthetic phenotype, confirming that Mg^2+^ and polyamines are required for the same process(es). Mutants lacking PaeA are also attenuated, but deleting *paeA* has no phenotype in a polyamine synthesis mutant background. These data support the idea that the cell coordinately controls both the polyamine and Mg^2+^ concentrations to maintain overall cation homeostasis, which is critical for survival in the macrophage phagosome.

## INTRODUCTION

Salmonella enterica serovars infect over 1 million people per year in the United States (1) and kill 355,000 people annually, worldwide (2). Lethal Salmonella infections result from the ability of the bacterium to survive and replicate in macrophages. Within the macrophage phagosome, Salmonella encounters a host-imposed Mg^2+^ limitation (3). Mg^2+^ is an essential divalent cation that is required to stabilize RNA and DNA (4–6), counteract the negative charges on adenosine triphosphate (ATP) (7), and neutralize the negative charges in outer membrane lipopolysaccharide (LPS) (8). Mg^2+^ is also critical for ribosome assembly, structure, and function (7, 9). In response to the low pH, Mg^2+^ limitation, and antimicrobial peptides in the phagosome, the PhoPQ signal transduction system induces the production of several factors required to adapt to this environment (10–12). Examples include the high-affinity Mg^2+^ transporters MgtA and MgtB (13, 14) as well as the enzymes that modify LPS, bypassing the need for Mg^2+^ stabilization (14, 15).

**FIG 1:**
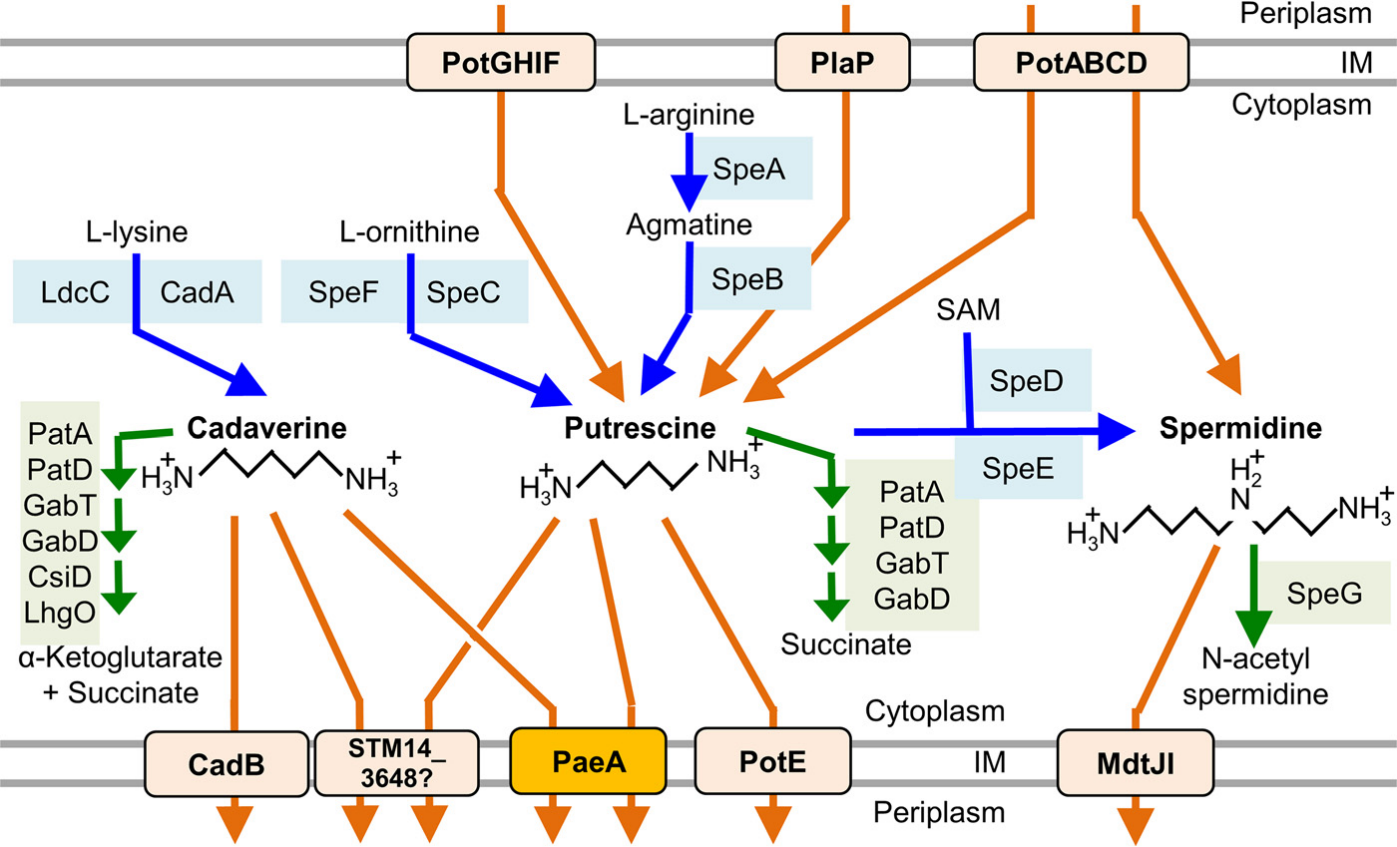
Known pathways of polyamine metabolism in Salmonella. The blue lines indicate synthetic pathways. The green lines indicate degradation or modification. The orange lines indicate transport. Mutant strains: Δdeg: Δ*patA* Δ*patD* Δ*csiD-R*; Δsynth: Δ*speA* Δ*speB* Δ*speC ΔspeF ΔspeDE ΔspeG ΔcadA Δldc*C. Modified from (33).

Polyamines are organic cations found in all three domains of life (16). However, the overall role of polyamines in the cell and their effects on Salmonella virulence are not understood. Salmonella and E. coli can synthesize the diamines cadaverine and putrescine as well as the triamine spermidine (17–19) (Fig. 1). In bacteria, polyamines are implicated in survival under anaerobic conditions, biofilm formation, swarming motility, and oxidative stress resistance (16, 17, 20, 21). It was also reported that putrescine enhances DNA supercoiling by activating DNA gyrase in Salmonella (22). Under mildly acidic conditions *in vitro*, the CadAB system is induced. CadA decarboxylates lysine to produce cadaverine, which consumes a proton and produces carbon dioxide. The cadaverine is secreted by the lysine:cadaverine antiporter CadB. This process helps to neutralize the cytoplasmic pH (19). The transcription of *cadAB* is activated by CadC in response to a low pH and the presence of lysine (23, 24). However, in the phagosome, OmpR represses the expression of *cadC* and *cadAB*, thereby allowing for the acidification of the cytoplasm, a condition required for the expression of virulence factors (25).

Polyamine cations bind to the same biomolecules as does Mg^2+^. It is estimated that over half of the polyamines in E. coli are found in complex with RNA (26) and that polyamines stabilize chromosome structure (22, 27). Biochemical analyses show that polyamines can also facilitate the assembly and function of ribosomal subunits (9, 28–30).

Both low and high polyamine concentrations are detrimental to E. coli and Salmonella, although the mechanisms are not known. Cells incapable of synthesizing polyamines have a mild growth defect under aerobic conditions but are incapable of growing anaerobically (17). Polyamines are also required in hyperbaric oxygen (20) and in several other specific conditions and genetic backgrounds (31). Salmonella strains that cannot synthesize putrescine or spermidine have been shown to be attenuated in several virulence assays (32). We have previously characterized the cadaverine and putrescine efflux protein PaeA. Salmonella and E. coli mutants lacking PaeA are killed by cadaverine or putrescine in the stationary phase, suggesting that high intracellular levels of polyamines are bactericidal (33). In addition, both low and high intracellular concentrations of polyamines are reported to affect the translation of discrete subsets of proteins (34, 35).

Here, we show that polyamines are critical for the adaptation of Salmonella to low Mg^2+^ conditions, including those found in the macrophage phagosome. Our overall results suggest that polyamines are synthesized upon low Mg^2+^ stress and partially replace Mg^2+^ until the cytoplasmic Mg^2+^ levels are restored. Indeed, our data show that it is the sum of Mg^2+^ and polyamines in the cell that is critical for viability. While Mg^2+^ and polyamines compensate for one another, too little of both or too much of both is lethal. PaeA, the expression of which is induced by PhoPQ in response to low Mg^2+^, is needed to efflux cadaverine and putrescine after the cytoplasmic Mg^2+^ levels are reestablished in order to avoid the toxic effect of excess divalent cations. Finally, our data show that both polyamine synthesis and efflux are required for full virulence in Salmonella.

## RESULTS

### PaeA is regulated by PhoPQ and is induced in response to low Mg^2+^ and during infection.

We previously showed that PaeA is responsible for cadaverine and putrescine efflux from the cell under certain conditions (33). To better understand the role of PaeA in Salmonella physiology and pathogenesis, we wanted to address the regulation of *paeA* expression. Transcriptomic data (36) suggests that *paeA* (*ytfL*) is expressed under most of the conditions that were tested, but is also significantly induced in macrophages. We used low pH (pH 5.6) and Mg^2+^ starvation to mimic the conditions that Salmonella encounters in macrophages to see whether PhoPQ, which controls the expression of numerous genes in the phagosome, has any role in the regulation of *paeA* expression.

**FIG 2:**
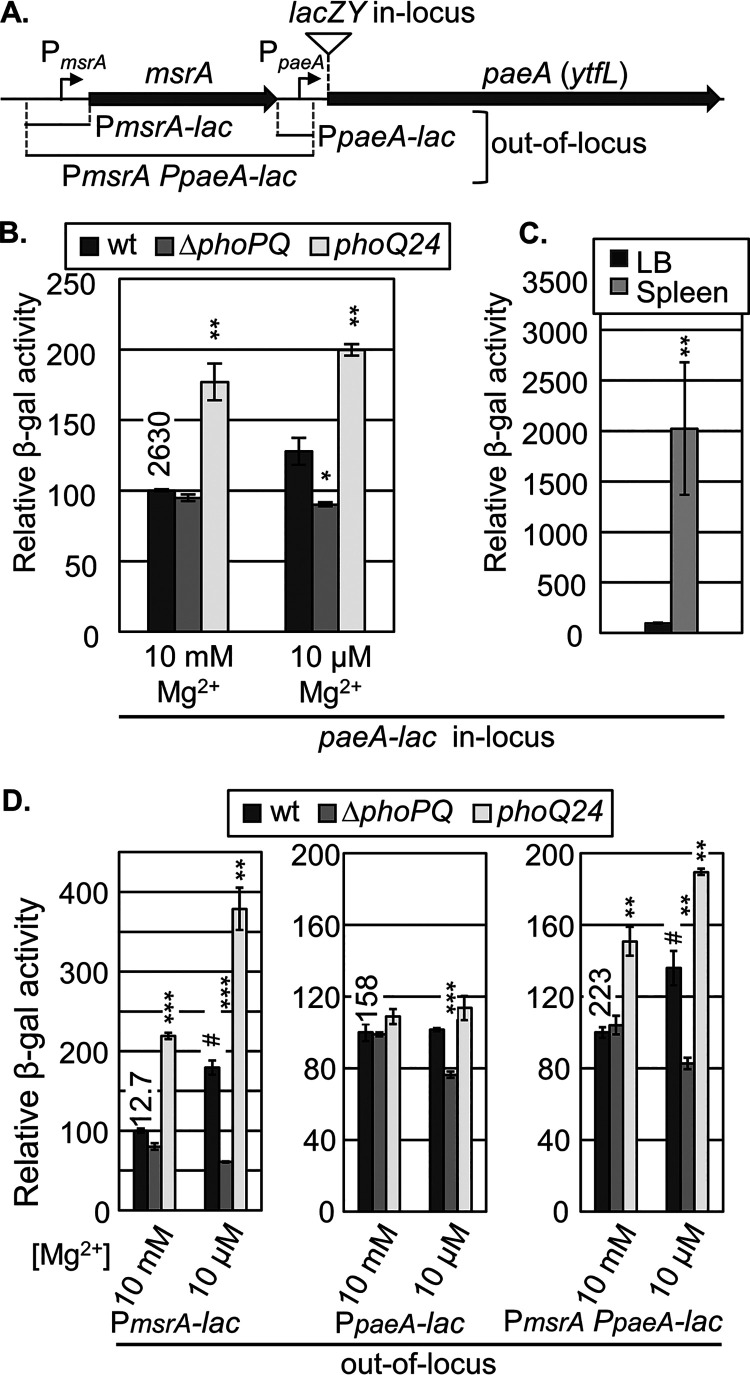
Expression of *paeA* is induced in response to low Mg^2+^ and in the mouse spleen. Regulation by PhoPQ requires an upstream P*msrA* promoter. (A) Diagram of the *msrA paeA* (*ytfL*) locus, showing the promoters and indicating both the in-locus and out-of-locus fusion constructs. (B) Strains containing the in-locus *paeA-lac* fusion in the *phoPQ*^+^ (wt), Δ*phoPQ*, and constitutive *phoQ*24 backgrounds were grown in N-minimal medium, pH 5.6, supplemented with 10 mM or 10 μM MgCl_2_. (C) BALB/c mice were infected with the *paeA-lac* in-locus transcriptional fusion strain. Bacteria were isolated from spleens 4 days postinfection or grown in LB medium for 16 h, and the β-galactosidase activity/CFU was determined via chemiluminescence assay for both. (D) Strains containing the out-of-locus P*msrA-lac*, P*paeA-lac*, or P*msrA* P*paeA-lac* fusion constructs in the indicated backgrounds were grown as described in panel B. (B and D) The relative β-galactosidase activity is a percentage of the value of the *phoP*^+^ strain grown in 10 mM Mg^2+^ (actual β-gal units for the control are indicated on the tops of the bars). *β*-gal activity is presented as the mean *±* SD, *n* *=* 3. Statistical analyses were performed using either an unpaired *t* test (**, *P* < 0.005; ***, *P* < 0.0005) versus the WT or a paired *t* test (^#^, *P* < 0.05) versus the different Mg^2+^ levels. (C) Relative β-galactosidase activity is a percentage of the value of the strain grown in LB. The *β*-gal activity/CFU is presented as the mean *±* SD, *n* *=* 5 (spleen), *n* = 3 (LB). Unpaired *t* tests were used. **, *P* < 0.005. Strains used: JS2601, JS2602, JS2603, JS2604, JS2605, JS2606, JS2607, JS2608, JS2609, JS2610, JS2611, JS2612, and JS2601.

Strains containing an in-locus single-copy chromosomal *paeA-lac* transcriptional fusion (Fig. 2A) were grown in pH 5.6 N-minimal medium that was either Mg^2+^ replete (10 mM Mg^2+^) or Mg^2+^ deficient (10 μM Mg^2+^). As shown in Fig. 2B, *paeA* expression was slightly induced in Mg^2+^-limited conditions, and this induction was dependent on PhoPQ. Expression was further induced in the *phoQ24* constitutive mutant background, independent of Mg^2+^ availability. These results show that *paeA* expression is controlled by the PhoPQ two-component regulatory system in response to host-like conditions. When the same in-locus *paeA-lac* fusion strain was used to infect BALB/c mice, the expression of *paeA* in bacteria isolated from infected mouse spleens was increased by 20-fold, compared to bacteria grown in LB medium (Fig. 2C).

Although regulated by PhoPQ, the expression of the *paeA* fusion was relatively high in the *phoP* null background. The published transcriptomic data indicate a primary *paeA* promoter upstream of the ORF (Fig. 2A) but suggest that there is readthrough from the upstream *msrA* promoter, particularly in the macrophage (36). We constructed several *lacZ* fusions by cloning the *msrA* promoter region, the *paeA* promoter region, and a fragment containing both promoters into the pDX1 expression vector (37), and we integrated these fusion constructs at the lambda attachment site (out-of-locus). The expression of the *paeA* promoter fusion was unaffected by low Mg^2+^ or by the *phoQ24* constitutive mutation (Fig. 2D). The loss of PhoPQ caused a slight decrease in *paeA* expression in the *paeA-lac* fusion strain for reasons that are not understood. In contrast, the *msrA* promoter fusion was clearly regulated by PhoPQ in response to low Mg^2+^. The fusion containing both promoters behaved similar to the in-locus *paeA-lac* fusion. These data suggest that *paeA* expression is controlled by its own promoter but is further induced under low Mg^2+^ conditions and, presumably, in the phagosome by readthrough from the *msrA* promoter, which is strongly regulated by PhoPQ.

### PaeA is required for survival under Mg^2+^ starvation.

**FIG 3:**
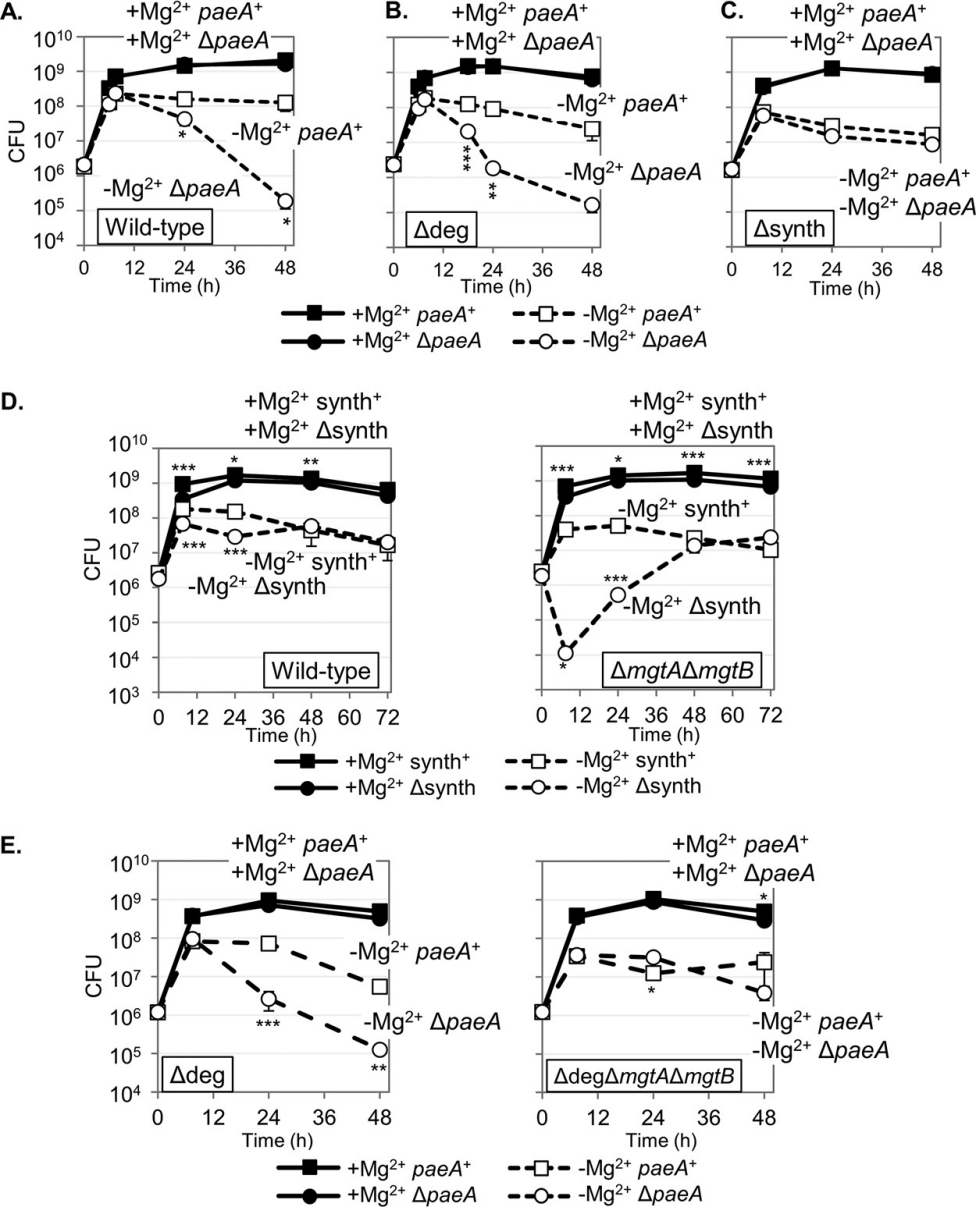
Survival under Mg^2+^ starvation conditions. The indicated strains, pregrown to the mid-exponential phase in N-minimal medium with 10 mM MgCl_2_, were washed, diluted into N-minimal medium with or without 10 mM MgCl_2_, and incubated at 37°C. CFU were determined at the indicated time points. Δdeg: Δ*patA* Δ*patD* Δ*csiD-R;* Δsynth: Δ*speA* Δ*speB* Δ*speC ΔspeF ΔspeDE ΔspeG ΔcadA ΔldcC.* CFU values are presented as the mean ± SD, *n* = 3 (A–C) or *n* = 6 (D, E). Statistical analysis was performed by using unpaired *t* tests of either (A–C, E) Δ*paeA* versus the corresponding *paeA*^+^ parent strain that received the same treatment or (D) Δsynth versus synth^+^ (*, *P* < 0.05; **, *P* < 0.005; ***, *P* < 0.0005). Strains used: 14028, JS2430, JS2464, JS2465, JS2560, JS2561, JS2562, JS2563, JS2564, and JS2565.

The *paeA* gene is partially regulated by PhoPQ and is induced during systemic infection. Therefore, we speculated that PaeA is important for growth or survival under Mg^2+^ deficient conditions. To test this hypothesis, we grew the wild-type and Δ*paeA* strains overnight in Mg^2+^ replete medium and then washed and diluted the cells into either Mg^2+^-replete or Mg^2+^-deficient medium. We monitored both the colony forming units (CFU) and the OD_600_ over a period of 48 h. As shown in Fig. 3A, the deletion of *paeA* had no effect on the initial growth under Mg^2+^-sufficient or Mg^2+^-deficient conditions, although the maximum number of CFU was reduced under Mg^2+^-limited conditions. However, in the stationary phase, the Δ*paeA* strain lost viability under Mg^2+^-deficient conditions, with the CFU being reduced by ~3 logs at 48 h. The OD_600_ was unaffected by the deletion of *paeA*, showing that the cells were not lysing under these conditions (Fig. S1A).

10.1128/mbio.02698-22.1 FIG S1 PaeA is required for survival under Mg^2+^ starvation conditions. (A) OD_600_ measurements for Fig. 3A, B, and C. The OD_600_ is presented as the mean ± SD, *n* = 3. Unpaired *t* test (*, *P* < 0.05; **, *P* < 0.005; ***, *P* < 0.0005) versus the parent strain that received the same treatment. (B) Survival of strains containing pWKS30-*paeA* or a vector plasmid after growth in Mg^2+^-replete or Mg^2+^-deficient conditions. CFUs were determined at 7.5 and 24 h. CFUs are presented as the mean ± SD, *n* = 3. Unpaired *t* test (*, *P* < 0.05; **, *P* < 0.005; ***, *P* < 0.0005) versus Δdeg at the same Mg^2+^ concentration. (C and D) The wild-type, Δ7 (Δ*cadB* Δ*potE* Δ*STM14_3648* Δ*speDE* Δ*patA* Δ*patD* Δ*csiD-R* Δ*paeA* Δ*mgtJI*), and indicated Δ6 strains when grown in (C) neutral N-minimal medium (pH 7.4) or (D) acidic N-minimal medium (pH 5.5) with or without 10 mM MgCl_2_. CFUs are presented as the mean ± SD, *n* = 6. Unpaired *t* test (*, *P* < 0.05; **, *P* < 0.005; ***, *P* < 0.0005) versus the Δ7 strain that received the same treatment or (lines) versus the wild-type that received the same treatment. Strains used: 14028, JS2430, JS2464, JS2465, JS2560, JS2561, JS2566, JS2567, JS2568, JS2569, JS2570, JS2571, JS2572, JS2573, JS2574, JS2575, JS2576, and JS2577. Download FIG S1, TIF file, 2.9 MB. Copyright © 2022 Iwadate et al. 2022 Iwadate et al. https://creativecommons.org/licenses/by/4.0/ This content is distributed under the terms of the Creative Commons Attribution 4.0 International license.

PaeA is a putative cadaverine and putrescine efflux pump (33). Therefore, we speculated that blocking polyamine degradation would enhance the *paeA* phenotype. To test this hypothesis, we created a degradation deficient strain (Δdeg) that lacks all of the genes involved in cadaverine and putrescine degradation (Fig. 1). As shown in Fig. 3B, the effect of deleting *paeA* on the survival under Mg^2+^-deficient conditions was slightly more pronounced in the Δdeg background. Indeed, there was a slight loss of viability in the Δdeg *paeA*^+^ strain, relative to that of the wild-type in low Mg^2+^. This finding is consistent with the role of polyamines in the phenotype. The introduction of a low copy number plasmid encoding PaeA (pWKS30-*paeA*) into the Δ*paeA* strain complemented the survival defect under Mg^2+^-deficient conditions (Fig. S1B). These results suggested that the PaeA-dependent efflux of cadaverine and putrescine is important for survival under Mg^2+^ starvation.

Since PaeA is responsible for cadaverine and putrescine efflux, the requirement for PaeA to maintain viability under Mg^2+^ starvation should depend on cellular polyamine synthesis. We constructed polyamine synthesis-deficient strains (Δsynth) that lacked all of the known genes for polyamine synthesis (Fig. 1), and we determined the effect of deleting *paeA* on survival under Mg^2+^ starvation conditions. As shown in Fig. 3C, the Δsynth strain grew to a slightly lower final CFU, particularly in low Mg^2+^. When *paeA* was deleted in this Δsynth background, there was no further loss of viability in the stationary phase in Mg^2+^-deficient medium (Fig. 3C), in contrast to when *paeA* was deleted in the wild-type background (Fig. 3A). Thus, the loss of viability in the Δ*paeA* strain under Mg^2+^ starvation is dependent on polyamine synthesis, showing that PaeA-dependent efflux decreases the toxic effect of endogenous polyamine production.

There are seven known or putative polyamine clearance pathways (Fig. 1): the cadaverine and putrescine efflux protein PaeA (33), the acid-inducible lysine:cadaverine antiporter CadB (38), the acid-inducible ornithine:putrescine antiporter PotE (39), the putative proteobacterial antimicrobial compound efflux (PACE) family cadaverine/putrescine pump STM14_3648 (40), the multidrug/spermidine efflux pump MgtJI (41), the synthesis pathway that makes spermidine from putrescine (SpeED) and potentially modifies spermidine (SpeG) (42, 43), and the cadaverine and putrescine degradation pathway PatA, PatD, CsiD-R (44, 45). To assess which pathways are important for survival under Mg^2+^-deficient conditions, we constructed a strain deleted for all seven pathways (the Δ7 strain) as well as strains that have only one of the pathways intact (the Δ6 strains). We determined the viability of each strain under Mg^2+^-replete and Mg^2+^-deficient conditions. As shown in Fig. S1C, only the wild-type and Δ6 (*paeA*^+^) strain maintained viability upon Mg^2+^ starvation, whereas the Δ7 strain and the other Δ6 strains that lack *paeA* lost viability. These results show that PaeA is the most important polyamine clearance system for survival under these Mg^2+^-deficient conditions.

The experiments above were performed in buffered pH 7.4 medium to avoid inducing the acid stress response, which is partially dependent upon polyamine synthesis and efflux (19). However, the macrophage phagosome is not only limited in Mg^2+^ but also acidic. Therefore, we investigated whether PaeA is important for survival in pH 5.5 media with or without Mg^2+^. As shown in Fig. S1D, not only the Δ6 (*paeA*^+^) strain but also the Δ6 (*cadB*^+^) strain maintained viability in Mg^2+^-deficient conditions. First, these results show that the PaeA-dependent efflux of cadaverine and/or putrescine is important for survival under Mg^2+^-limited conditions in both acidic and neutral environments. Second, these results are consistent with the CadAB system being induced in low pH, leading to the production of cadaverine from lysine by CadA and export by the lysine:cadaverine antiporter CadB as a mechanism to maintain the cytoplasmic pH (19). Thus, if it is expressed, CadB can play an important role in polyamine efflux under low Mg^2+^ stress. Interestingly, polyamine efflux systems are dispensable under acidic conditions when Mg^2+^ levels are sufficient (Fig. S1D).

### Polyamine synthesis is required for growth and survival during Mg^2+^ starvation in the absence of inducible Mg^2+^ transporters.

The data presented above suggest that polyamine synthesis is induced in response to low Mg^2+^ and that PaeA, which is also induced under low Mg^2+^ conditions, is required to export the polyamines in order to prevent a loss of viability late in the response. We hypothesize that Salmonella induces polyamine synthesis and that these organic cations compensate for low intracellular Mg^2+^. However, deletion of known polyamine synthesis genes in the wild-type background confers only a minor defect in growth or survival under Mg^2+^ starvation (compare Fig. S2A and C).

10.1128/mbio.02698-22.2 FIG S2 Putrescine and spermidine synthesis is critical for growth and survival during Mg^2+^ starvation in the absence of inducible Mg^2+^ transporters. The indicated strains were grown in N-minimal medium with or without 10 mM MgCl_2_ as in Fig. 2, and CFUs were determined at 0 and 7.5 h. CFUs are presented as the mean ± SD, *n* = 3. Unpaired *t* test (*, *P* < 0.05; **, *P* < 0.005; ***, *P* < 0.0005) versus the parent vector strain that received the same treatment. Strains used: JS2452, JS2584, JS2585, JS2586, JS2587, JS2588, JS2589, JS2590, JS2591, and JS2592. Download FIG S2, TIF file, 2.2 MB. Copyright © 2022 Iwadate et al. 2022 Iwadate et al. https://creativecommons.org/licenses/by/4.0/ This content is distributed under the terms of the Creative Commons Attribution 4.0 International license.

Salmonella has three Mg transporters: CorA, MgtA, and MgtB. Under Mg^2+^-replete conditions, Mg^2+^ is transported primarily through CorA. However, CorA is known to be inactivated once cytoplasmic Mg^2+^ levels drop (46). Under these conditions, MgtA and MgtB are induced and serve as the primary Mg^2+^ transporters in the cell. We investigated whether polyamine synthesis is required for survival under Mg^2+^ starvation when the two inducible Mg^2+^ transporters are absent. As shown in Fig. 3D, in the absence of polyamine synthesis, the strain lacking both inducible Mg^2+^ transporters lost about 2 logs of viability in 6 h and then slowly grew to the level of the other strains. This is a reproducible phenomenon, but whether this is an adaptation or the result of suppressor mutations remains to be determined. A low copy number plasmid encoding both MgtA and MgtB (pWKS30-*mgtA-mgtB*) complemented the survival defect under Mg^2+^ deficient conditions in the Δ*mgtA* Δ*mgtB* Δsynth strain (Fig. S2). These results show that either polyamine synthesis or Mg^2+^ transport is required for growth and survival under Mg^2+^ starvation conditions and suggest that polyamines and Mg^2+^ support the same biological process(es).

### The PaeA-mediated efflux of polyamines is required only when Mg^2+^ is imported.

PaeA-dependent polyamine efflux is required for survival at the late stage of Mg^2+^ starvation (Fig. 3A). Our model, previous data (33), and the data presented above suggest that this lethality is caused by an excess of Mg^2+^ and polyamines under these conditions. To further test this hypothesis, we asked whether PaeA is still required for survival at the late stage of Mg^2+^ starvation when the strain lacks the two inducible Mg^2+^ transporters MgtA and MgtB. As shown in Fig. 3E, the loss of PaeA in the Δ*mgtA* Δ*mgtB* background had no effect on viability in the stationary phase. Thus, PaeA-dependent polyamine efflux is no longer required for survival when Mg^2+^ is not imported by the two inducible Mg^2+^ transporters MgtA and MgtB, implying that polyamines are not toxic when cytoplasmic Mg^2+^ levels stay below some threshold. Indeed, polyamines are required under these conditions.

**FIG 4:**
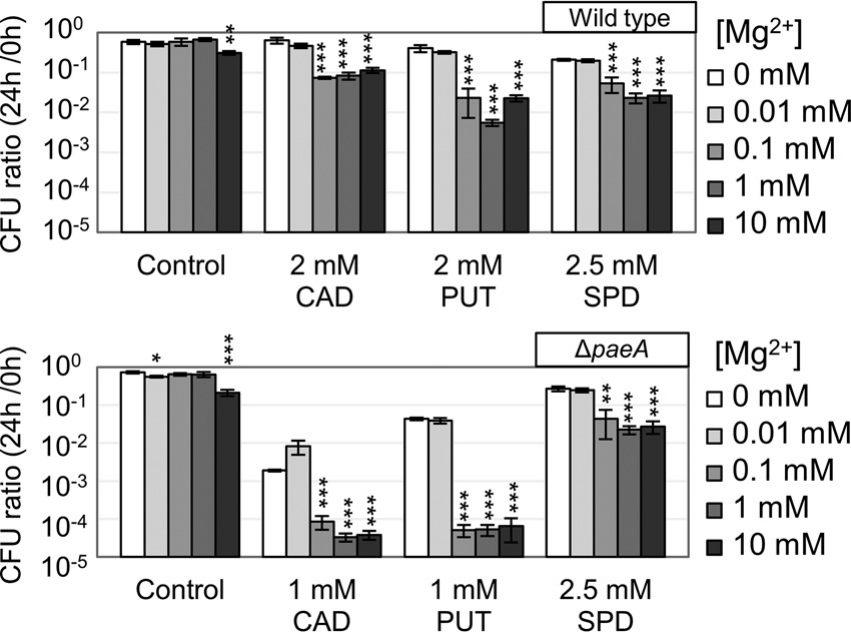
Higher Mg^2+^ concentrations sensitize cells to polyamines. The strains were grown in N-minimal medium with 0, 0.01, 0.1, 1, or 10 mM MgCl_2_ for 4 h at 37°C, washed, diluted into pH 8.5-buffered saline containing the indicated polyamines, and incubated at 37°C. CAD, cadaverine; PUT, putrescine; SPD, spermidine. CFU were determined at 0 and 24 h. CFU values are presented as the mean ± SD, *n* = 6. Statistical analysis was performed via paired *t* tests (*, *P* < 0.05; **, *P* < 0.005; ***, *P* < 0.0005) of a 10 mM MgCl_2_ concentration versus a 0 mM MgCl_2_ control. Strains used: 14028 and JS2430.

We initially identified PaeA as conferring cadaverine and putrescine resistance to stationary phase cells incubated in a high pH buffer (33). To further examine the correlation between polyamine toxicity and Mg^2+^ levels, we determined the polyamine tolerance in cells initially grown in different Mg^2+^ concentrations. As shown in Fig. 4, when cells were grown in medium containing only the Mg^2+^ found in the medium components (0 μM added MgCl_2_) or in medium with 0.01 mM of added MgCl_2_, both the wild-type and Δ*paeA* strains showed higher tolerances to cadaverine, putrescine, and spermidine than did cells grown with 0.1 mM or higher concentrations of added MgCl_2_. Sensitivity to cadaverine and putrescine was greatly enhanced in the absence of PaeA, whereas sensitivity to spermidine was unchanged, as we previously reported (33). Collectively, these results are consistent with our model suggesting that cadaverine and putrescine levels become toxic only when Mg^2+^ levels exceed some threshold.

### Putrescine and spermidine are the most important polyamines under Mg^2+^ limitation.

**FIG 5:**
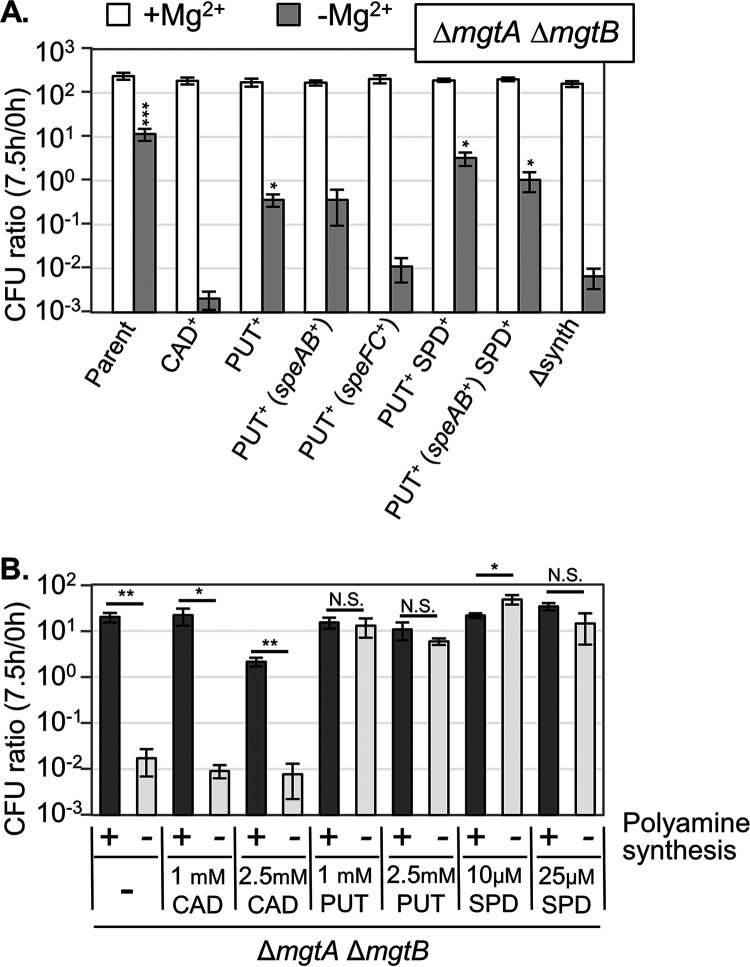
Putrescine and spermidine are critical for survival during Mg^2+^ starvation in the absence of inducible Mg^2+^ transporters. (A) The Δ*mgtA* Δ*mgtB* strains capable of synthesizing the indicated polyamines were grown as in Fig. 3, and CFU were determined at 0 and 7.5 h. CAD, cadaverine; PUT, putrescine; and SPD, spermidine. (B) The Δ*mgtA* Δ*mgtB* and Δ*mgtA* Δ*mgtB* Δsynth strains were pregrown to the mid-exponential phase in N-minimal medium with 10 mM MgCl_2_, washed, diluted into N-minimal medium with 0 mM MgCl_2_ and the indicated polyamines, and incubated at 37°C. CFU were determined at 0 and 7.5 h. Note that SPD is inhibitory at higher concentrations. CFU values are presented as the mean ± SD, *n* = 6. Statistical analyses were performed via (A) Unpaired *t* test (*, *P* < 0.05; **, *P* < 0.005; ***, *P* < 0.0005) versus the Δsynth strain. (B) Unpaired *t* test (*, *P* < 0.05; **, *P* < 0.005; ***, *P* < 0.0005) of the Δsynth strain versus the synth^+^ strain under each condition. Strains used: JS2562, JS2578, JS2579, JS2580, JS2581, JS2582, JS2583, and JS2563.

To clarify which polyamine is most important for growth and survival under Mg^2+^ starvation conditions, we constructed strains that synthesize only cadaverine, only putrescine, or both putrescine and spermidine, all in a Δ*mgtA* Δ*mgtB* background. Note that spermidine is synthesized from putrescine, so we cannot make a strain that synthesizes only spermidine (Fig. 1). As shown in Fig. 5A, the strains that can synthesize putrescine and spermidine survived nearly as well as did the parent strain (Δ*mgtA* Δ*mgtB*) under Mg^2+^-limited conditions at 7.5 h. In contrast, the strain that synthesized only cadaverine showed similar CFU to those of the Δ*mgtA* Δ*mgtB* Δsynth strain. These results suggest that putrescine and spermidine are the most important polyamines for survival under Mg^2+^ starvation conditions.

Salmonella has three independent putrescine synthesis pathways: SpeAB, SpeC, and SpeF (Fig. 1). To determine which pathways are responsible for survival under Mg^2+^ starvation, we created strains that have only the SpeAB pathway or both the SpeC and SpeF pathways, and we determined the survival under Mg^2+^ starvation conditions. As shown in Fig. 5A, the SpeC and SpeF putrescine synthesis pathways were dispensable, whereas the SpeAB pathway allowed for survival under Mg^2+^ starvation. These results suggest that the SpeAB pathway for putrescine synthesis and the SpeED pathway for spermidine synthesis are the most important for survival under Mg^2+^ starvation. To confirm these results, we performed a series of complementation tests. When a low copy number plasmid encoding SpeA, SpeB, SpeE, and SpeD was introduced into the Δ*mgtA* Δ*mgtB* Δsynth strain, the survival under Mg^2+^-deficient conditions was restored almost to the level of the parent Δ*mgtA* Δ*mgtB* strain (Fig. S2). These results confirm that the putrescine and spermidine synthesis pathways are sufficient for the survival of the Δ*mgtA* Δ*mgtB* strain under Mg^2+^ starvation.

The results presented above show that the endogenous production of spermidine and/or putrescine is sufficient to protect the cell during Mg^2+^ starvation. We then investigated whether exogenous polyamines could also confer protection under these conditions. As shown in Fig. 5B, the addition of putrescine or spermidine to the medium prevented the loss of viability of the Δ*mgtA* Δ*mgtB* Δsynth strain upon a transition to Mg^2+^-deficient conditions. Note that these cells are incapable of making spermidine from putrescine or of modifying the putrescine that was added. It should also be noted that there is no known cadaverine importer in Salmonella, so it is not clear whether cadaverine is incapable of rescuing the phenotype or whether cadaverine simply did not enter the cell in any significant concentration. These results show that putrescine and spermidine are independently capable of protecting cells from a loss of viability during Mg^2+^ starvation in the absence of inducible Mg^2+^ transporters.

### Polyamine and Mg^2+^ levels are coordinately regulated.

**FIG 6:**
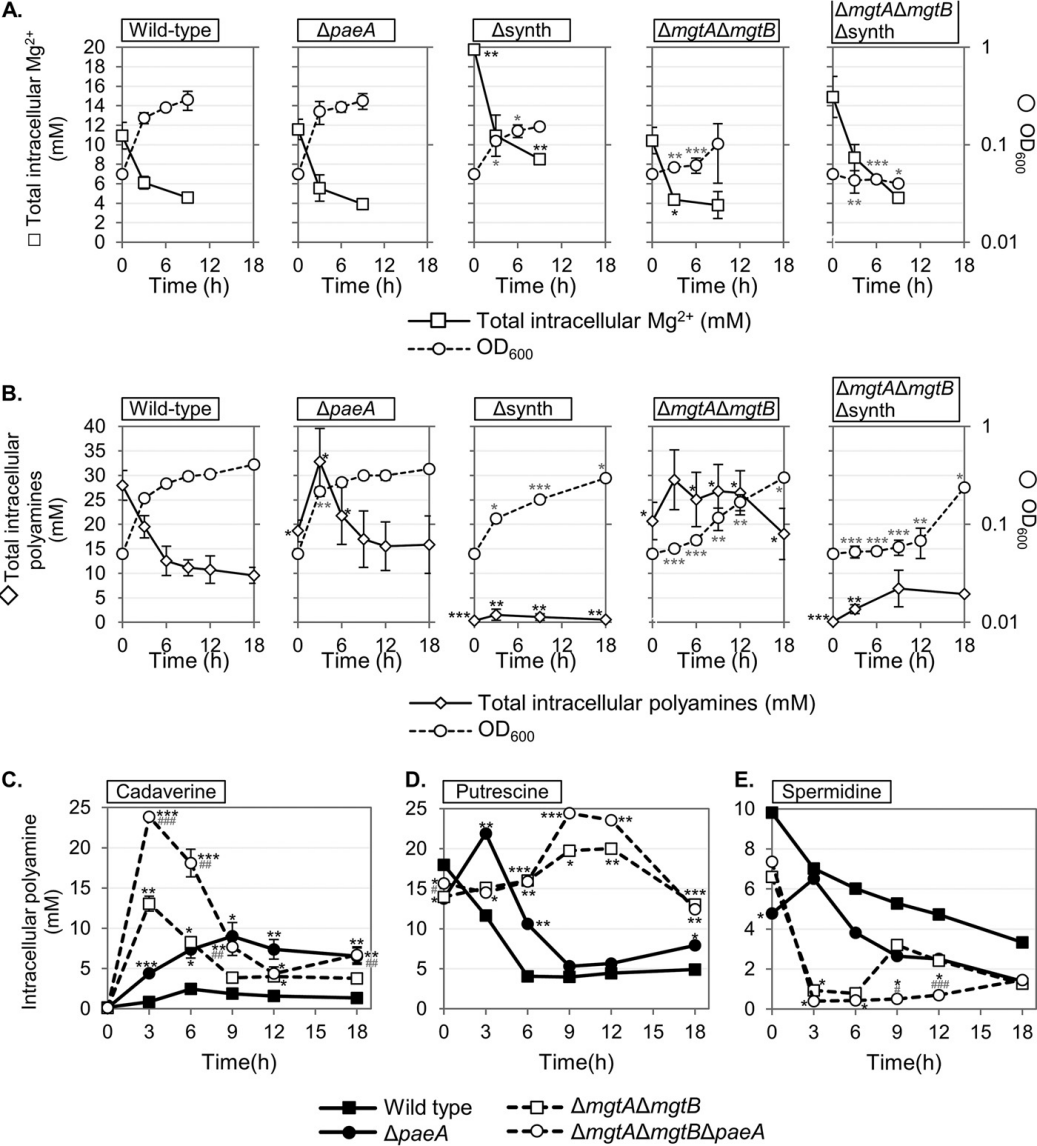
Cellular polyamine levels and Mg^2+^ levels are inversely correlated. The indicated strains were pregrown to the mid-exponential phase in N-minimal medium with 10 mM MgCl_2_, washed, diluted into N-minimal medium without MgCl_2_, and incubated at 37°C. Intracellular polyamine and Mg^2+^ levels were measured at the indicated time points. (A) Left axis, intracellular Mg^2+^ concentrations; right axis, OD_600_. (B) Left axis, total intracellular polyamine (cadaverine, putrescine, and spermidine) concentrations; right axis, OD_600_. (C–E) Indicated intracellular polyamine concentrations. The concentrations and the OD_600_ are presented as the mean ± SD, *n* = 3. Statistical analyses were performed via (A and B) Unpaired *t* test (*, *P* < 0.05; **, *P* < 0.005; ***, *P* < 0.0005) versus the wild-type concentration (in black) or the wild-type OD_600_ (in gray) at the same time point; (C–E) Unpaired *t* test (*, *P* < 0.05; **, *P* < 0.005; ***, *P* < 0.0005) versus the wild-type concentration at the same time point or (^#^, *P* < 0.05; ^##^, *P* < 0.005; ^###^, *P <* 0.0005) of Δ*paeA* versus the corresponding *paeA*^+^ strain at the same time point. Strains used: 14028, JS2430, JS2560, JS2562, JS2563, and JS2598.

Our results suggest that the Mg^2+^ and polyamine levels in the cell are coordinately regulated. We measured intracellular polyamines and Mg^2+^ in the wild-type and in various mutant backgrounds upon shifting the cells to Mg^2+^-depleted conditions. As seen in Fig. 6A and B, the wild-type cells continued to grow exponentially until 3 h after the media shift (OD_600_, right axis). The initial intracellular concentration of Mg^2+^ in the wild-type cells decreased from approximately 11 mM to approximately 5 mM over 9 h, whereas the total polyamine concentration dropped from approximately 27 mM to 10 mM at 18 h. Note that the cells continued to divide, explaining some of the decrease in cellular concentrations. As expected, the loss of PaeA resulted in higher intracellular levels of polyamines, compared to the wild-type, over the course of the experiment, peaking at 3 h after the media shift, whereas the Mg^2+^ concentrations were similar to those of the wild-type. Note that the *paeA* mutant was likely starting to die after 12 h (Fig. 3A; note that the OD_600_, not the CFU, was measured in all of the experiments in Fig. 6).

We observed mutually compensating changes in the intracellular concentrations of Mg^2+^ and polyamines when the cells were depleted of Mg^2+^. The loss of polyamine synthesis resulted in higher intracellular Mg^2+^ levels, whereas the loss of Mg^2+^ transporters resulted in increased intracellular polyamine levels. The Δsynth mutant started with approximately 20 mM Mg^2+^, twice the level of the wild-type, suggesting that the cells were compensating for the loss of polyamines prior to being shifted to the no-Mg^2+^ medium. Moreover, the Mg^2+^ levels remained significantly higher than those of the wild-type over the course of the measurement. In the Δ*mgtA* Δ*mgtB* strain, the Mg^2+^ concentrations were slightly lower than those of the wild-type, and the polyamine concentrations remained high. Finally, in the Δ*mgtA* Δ*mgtB* Δsynth strain, the Mg^2+^ concentrations were initially higher than those in the wild-type, but they decreased over 9 h. Note that these cells were likely losing viability over this time. Overall, these results are consistent with the cells maintaining a reciprocal relationship between the Mg^2+^ and polyamine concentrations. Our model suggests that the Δ*paeA* cells are dying because the Mg^2+^ plus polyamine concentrations are too high, whereas the Δ*mgtA* Δ*mgtB* Δsynth mutant is dying because the combined concentrations are too low.

We also determined the concentrations of the individual polyamines in the wild-type and Δ*mgtA* Δ*mgtB* strains as well as the effect of the *paeA* deletion on these levels. As shown in Fig. 6C, the wild-type cells had low levels of cadaverine. The levels were increased in the *paeA* mutant, as expected. The deletion of the Mg^2+^ transporters led to a dramatic increase in the cadaverine concentration at 3 h after the shift, with a subsequent decrease being observed over time. The putrescine levels (Fig. 6D) were approximately 18 mM when the cells were shifted to the no-Mg^2+^ medium and decreased in the wild-type. They remained high initially in the *paeA* mutant but then decreased. In the absence of the Mg^2+^ transporters, the putrescine levels had increased from 9 to 12 h, as opposed to the decrease seen in the wild-type background. For both cadaverine and putrescine, the simultaneous loss of PaeA and the Mg^2+^ transporters resulted in a further increase in intracellular levels.

The spermidine levels were generally lower in all strains and decreased inversely with cadaverine or putrescine, including in the *paeA* mutant backgrounds (Fig. 6E). Indeed, it is possible that the putrescine levels are partially maintained by decreasing the synthesis of spermidine. Together, these data support a model in which synthesis of putrescine and cadaverine is increased in response to low Mg^2+^, but it does appear that production of the two polyamines is differentially regulated.

### Polyamine synthesis and efflux are important for Salmonella virulence.

To examine whether the phenomena observed *in vitro* are relevant *in vivo*, we performed competition assays after intraperitoneal infection (IP) in BALB/c mice. As shown in Table 1, the *paeA* mutant was approximately 2.3-fold attenuated, relative to the isogenic wild-type strain, and this difference was statistically significant. BALB/c mice are susceptible to Salmonella infections due to a mutation in Nramp1 (Slc11a1) (47), a divalent cation transporter in the phagosomal membrane (48). Recently, it was reported that Nramp1 restricts Salmonella by reducing the Mg^2+^ availability (3). Therefore, we also used C3H mice, which have a functional Nramp1 (49), to test the effect of the *paeA* deletion on virulence. As shown in Table 1, the *paeA* mutant was attenuated >6-fold in the C3H mice. This result shows that loss of PaeA confers a virulence defect and this phenotype is exacerbated in the C3H mice, which are presumed to have lower Mg^2+^ concentrations in the phagosome. Next, we tested whether polyamine synthesis is required during infection. As shown in Table 1, the polyamine synthesis mutant was 500-fold attenuated, relative to the wild-type, in the C3H mice. As expected, the deletion of *paeA* in the synthesis mutant conferred no phenotype relative to the Δsynth strain in a competition assay. This result indicates that PaeA is effluxing endogenously produced polyamines *in vivo*. Next, to see which polyamine is the most important for virulence, we used strains that can make only cadaverine (CAD^+^), only putrescine (PUT^+^), or putrescine and spermidine (PUT^+^SPD^+^), with each competing against the isogenic wild-type strain. As shown in Table 1, both the PUT^+^ and PUT^+^SPD^+^ strains competed evenly with the wild-type strain. The CAD^+^ strain showed decreased competitive fitness, which is consistent with published results (32), but this strain was not attenuated to the level of the Δsynth strain, which has never been previously tested. These results suggest that putrescine is the most important polyamine for Salmonella virulence, and this is consistent with our *in vitro* results.

**TABLE 1:** PaeA is important for the virulence of Salmonella

Bacterial strains^*a*^						
Strain A	Strain B	Mouse strain	Median CI^*b*^	No. of mice	*P* value^*c*^	Fold attenuation^*d*^
Δ*paeA*	WT	BALB/c	0.44	13	0.02	2.3
Δ*paeA*	WT	C3H	0.16	10	<0.0005	6.3
Δsynthesis	WT	C3H	0.002	12	<0.0005	500.0
ΔsynthΔ*paeA*	Δsynth	C3H	0.84	6	N.S.	1.2
PUT+	WT	C3H	0.71	6	N.S.	1.4
CAD+	WT	C3H	0.12	6	0.001	8.3
PUT+SPD+	WT	C3H	1.38	6	N.S.	0.7
Δ*mgtA*Δ*mgtB*	WT	C3H	0.0000041	6	<0.0005	2.4× 10^5^
Δ*mgtB*	WT	C3H	0.059	6	<0.0005	16.9^*e*^
ΔsynthΔ*mgtB*	Δsynth	C3H	0.0098	6	<0.0005	102.0^*e*^

a The strains used were 14028 (WT), JS2430, JS2560, JS2561, JS2593, JS2594, JS2595, JS2562, JS2596, and JS2597.

b Bacteria were recovered from the spleen after intraperitoneal (i.p.) competition assays. The competitive index (CI) was calculated as (percent strain A recovered/percent strain B recovered)/(percent strain A inoculated/percent strain B inoculated).

c Unpaired Student’s *t* tests were used to compare the logarithmically transformed CI values to the inocula. N.S., not significant.

d The fold attenuation is 1/CI, reflecting the level of attenuation of strain A relative to strain B.

e Unpaired Student’s *t* test confirms that the Δ*mgtB* vs WT and ΔsynthΔ*mgtB* vs Δsynth groups differ at a *P* value of <0.005.

Next, we examined the relationship between polyamines and Mg^2+^ transport. As shown in Table 1, the Δ*mgtA* Δ*mgtB* strain was dramatically attenuated, relative to the wild-type, and this finding is consistent with previous reports (3, 50). To lessen this attenuation, we deleted *mgtB*, leaving *mgtA* intact. The Δ*mgtB* strain was 17-fold attenuated, relative to the wild-type. Then, we deleted *mgtB* in the Δsynth strain. The Δ*mgtB* Δsynth strain was 100-fold attenuated, relative to the Δsynth strain. Thus, these mutations show a synthetic phenotype that is consistent with the model, which asserts that polyamine synthesis compensates for low cytoplasmic Mg^2+^ during infection.

## DISCUSSION

**FIG 7:**
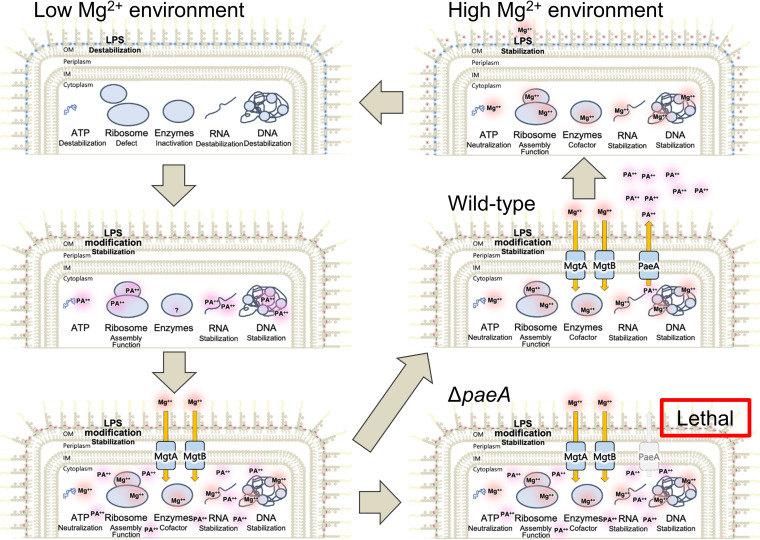
Model for Salmonella adaptation to low Mg^2+^. Mg^2+^ is required for the stabilization and/or function of LPS, ATP, RNA, DNA, ribosomes, and certain enzymes. Upon Mg^2+^ starvation, polyamine synthesis is induced by mechanisms that are not understood. The PhoPQ two-component regulatory system also responds to low Mg^2+^ by inducing the high-affinity Mg^2+^ transporters MgtA and MgtB. Either polyamine synthesis or high-affinity Mg^2+^ transport is sufficient for Salmonella to survive at the early stage of Mg^2+^ starvation. However, a mutant defective in both loses viability upon a shift to a no-Mg^2+^ medium. PaeA is also induced by PhoPQ. After Mg^2+^ levels are reestablished, PaeA is required to efflux polyamines. Otherwise, the cells lose viability when the combined concentration of polyamine and Mg^2+^ ultimately becomes too high.

We have shown that polyamine synthesis and efflux are required for growth and survival under low Mg^2+^ stress and are critical for Salmonella pathogenesis. The combined concentrations of the divalent cations, Mg^2+^ and polyamines, must be coordinately regulated; concentrations that are either too low or too high are lethal to the cell. Our data support the following model (Fig. 7). Upon Mg^2+^ starvation, polyamine synthesis is induced by mechanisms that are not understood. The PhoPQ two-component system also responds to low Mg^2+^ by inducing the high-affinity Mg^2+^ transporters MgtA and MgtB. In the laboratory, either polyamine synthesis or high-affinity Mg^2+^ transport is sufficient for Salmonella to survive Mg^2+^ starvation conditions. However, a mutant defective in both loses viability upon a shift to a no-Mg^2+^ medium. When both systems are intact, it appears that the combined polyamine and Mg^2+^ levels ultimately become too high, and PaeA, which is also induced by PhoPQ, is required to efflux polyamines. Otherwise, the cells lose viability. Consistent with this model, the loss of either polyamine synthesis or high-affinity Mg^2+^ transport suppresses the need for PaeA. Moreover, increasing Mg^2+^ concentrations in the medium exacerbates sensitivity to polyamines.

Importantly, these phenotypes are largely recapitulated during infection. Polyamine synthesis is important for Salmonella virulence (32). A strain incapable of making any polyamines is dramatically attenuated (Table 1). High affinity Mg^2+^ transport is also important for replication in the host (3, 50). Moreover, this requirement is significantly exacerbated in the absence of polyamine synthesis, which is consistent with a compensatory role for polyamines and Mg^2+^ during adaptation to the low Mg^2+^ environment of the macrophage phagosome. In an otherwise wild-type background, PaeA is required for full virulence, but is fully dispensable in the polyamine synthesis mutant, showing that endogenously produced polyamines can lead to decreased survival in the host if they are not effluxed from the cell. Moreover, the *paeA* phenotype is exacerbated in Nramp^+/+^ mice, which is consistent with increased polyamine synthesis in response to more stringent Mg^2+^ sequestration in the phagosome (3, 51).

Mutants lacking both high-affinity Mg^2+^ transporters and the ability to synthesize polyamines lose viability immediately upon a transition to a medium lacking Mg^2+^. Some essential process becomes dysfunctional in the Δ*mgtA* Δ*mgtB* Δsynth strain under low Mg^2+^ stress. We speculate that an acute drop in intracellular Mg^2+^ in the absence of polyamines primarily affects translation. Numerous reports suggest that Mg^2+^ and polyamine levels affect ribosome homeostasis. Previous studies have shown that ~80% of the Mg^2+^ associated with ribosomes can be functionally replaced by putrescine and spermidine (9, 30). Tabor and colleagues also showed that polyamines are essential for growth in an E. coli strain harboring an *rpsL* mutation (31), which confers streptomycin resistance and higher fidelity translation (52). They further showed that polyamines are essential for growth in a background lacking MnmEG, which modifies the wobble position U in the anticodon of a number of tRNAs (53). E. coli strains lacking putrescine are less sensitive to aminoglycosides and amino acid starvation, which is consistent with altered ribosome structure and function (54, 55). In addition to these general effects on translation, either low or high polyamine concentrations affect the translation of specific mRNAs, many of which encode important regulatory proteins, such as Fis, H-NS, CpxR, and RpoE (34, 35). How decreases in these specific proteins might affect the phenotypes that we have observed remains to be determined.

While the absence of polyamines leads to immediate lethality under low Mg^2+^ conditions, high levels of polyamines and Mg^2+^ seem to be lethal only in the stationary phase, despite the fact that polyamine levels in the *paeA* mutant peak as the cells enter the stationary phase (Fig. 6B). Our previous data suggested that high intracellular levels of cadaverine that last for less than 3 h resulted in death over 24 h. Thus, a spike in polyamine (and Mg^2+^) concentration could trigger a lethal event from which the cell cannot recover. More work is required to understand the lethality caused by either low or high polyamine and Mg^2+^ concentrations.

Putrescine and spermidine seem to be most critical for survival under low Mg^2+^ conditions and in the animal, despite their difference in charge. The cell has transporters for these compounds, and the addition of either suppresses lethality under low Mg^2+^ conditions, even in a strain incapable of converting putrescine to spermidine or modifying spermidine (Fig. 5B). Cadaverine, on the other hand, seems most critical for the adaptation to low pH conditions (19), and Salmonella has no mechanism to import cadaverine from the environment. However, a strain that can produce cadaverine is significantly less attenuated than a strain that cannot make any polyamines (Table 1), even though *cadAB* is not fully induced in the phagosome, which thereby maintains a low pH in the cytosol (25). Note also that CadB is dispensable under acidic conditions when Mg^2+^ levels are sufficient (Fig. 3D), suggesting that cadaverine synthesis is indeed coordinated with Mg^2+^ levels. It makes intuitive sense that putrescine and cadaverine would be interchangeable as simple divalent cations. However, the cell has regulatory mechanisms that independently control their synthesis and import. We do not understand the mechanisms by which the cell coordinates Mg^2+^ and polyamine levels, but several published results are consistent with this conclusion. For example, a mutant that cannot synthesize putrescine and spermidine shows increased levels of *mgtA* and *mgtB* transcription (56).

The fact that PaeA expression is induced under low Mg^2+^ conditions, despite polyamines being required for adaptation, is counterintuitive. It implies that PaeA might be controlled at the protein level such that cadaverine and putrescine are exported only after Mg^2+^ levels reach a certain threshold. For another example, upon activation, PhoP represses the expression of the ClpS adaptor protein for the ClpAP protease, which results in the stabilization of PatA, encoding the first enzyme in cadaverine and putrescine degradation (57). This should result in a decrease in polyamines, seemingly when they are most needed. Both PaeA and PatA likely contribute to polyamine-Mg^2+^ homeostasis, but the timing must be critical, and there are likely multiple levels of regulation.

In summary, we show that the cell coordinately regulates polyamine and Mg^2+^ concentrations and that total concentrations of the two are critical for cell viability. There are certainly instances in which Mg^2+^ (dehydrated) is specifically coordinated and is required for enzymatic function. Likewise, there might be functions that specifically require one of the polyamines. However, polyamines seem to primarily play the roles of simple cations that are capable of replacing Mg^2+^ (hydrated) in fundamental cell processes. It is likely that this general concept holds true in all cells.

## MATERIALS AND METHODS

### Bacterial strains and media.

The strains used are described in Table S1, while the plasmids used are listed in Table S2. All of the Salmonella strains used are derivatives of Salmonella enterica serovar Typhimurium strain 14028. Deletions with concomitant insertions of antibiotic resistance cassettes were constructed using λ Red-mediated recombination as previously described (58, 59), with the indicated endpoints (Table S1). The deletions were verified via polymerase chain reaction (PCR) analysis and were then transduced into a clean background using phage P22 HT105/1 int-201 (60). Antibiotic resistance cassettes were removed using the pCP20 plasmid. The plasmid construction is described in the supplemental material (Text S1). All of the plasmids were passaged through a restriction-minus modification-plus Pi^+^ Salmonella strain JS198 (59) prior to their transformation into Salmonella strains.

10.1128/mbio.02698-22.4 TABLE S1 Strain list. Download Table S1, DOCX file, 0.05 MB. Copyright © 2022 Iwadate et al. 2022 Iwadate et al. https://creativecommons.org/licenses/by/4.0/ This content is distributed under the terms of the Creative Commons Attribution 4.0 International license.

10.1128/mbio.02698-22.5 TABLE S2 Plasmid list. Download Table S2, DOCX file, 0.04 MB. Copyright © 2022 Iwadate et al. 2022 Iwadate et al. https://creativecommons.org/licenses/by/4.0/ This content is distributed under the terms of the Creative Commons Attribution 4.0 International license.

10.1128/mbio.02698-22.7 TEXT S1 Plasmid construction. Download Text S1, DOCX file, 0.03 MB. Copyright © 2022 Iwadate et al. 2022 Iwadate et al. https://creativecommons.org/licenses/by/4.0/ This content is distributed under the terms of the Creative Commons Attribution 4.0 International license.

The transcriptional in-locus *paeA*-*lac* fusion was constructed as described by Ellermeier et al. (59). The fusion joint is indicated in Table S1. Additional out-of-locus transcriptional fusions were constructed by cloning the following fragments upstream of the promotorless *lacZ* in the pDX1 vector and integrating the resulting plasmids in single copy at the *λ*-attachment site in the Salmonella chromosome as previously described (37): (i) *msrA* promoter only, (ii) *paeA* promoter only, and (iii) a region that includes the *msrA* promoter, ORF, and *paeA* promoter (Fig. 1A). The primers used and the endpoints of the cloned fragments are indicated in Tables S3 and S2, respectively.

10.1128/mbio.02698-22.6 TABLE S3 Primer list. Download Table S3, DOCX file, 0.04 MB. Copyright © 2022 Iwadate et al. 2022 Iwadate et al. https://creativecommons.org/licenses/by/4.0/ This content is distributed under the terms of the Creative Commons Attribution 4.0 International license.

During the construction of strains, cells were routinely grown at 37°C in Luria-Bertani (LB) medium containing 1% NaCl. Strains containing the temperature-sensitive plasmids pCP20 and pKD46 were grown at 30°C. Antibiotics were used as follows: ampicillin, 50 μg/mL; chloramphenicol, 10 μg/mL; kanamycin, 50 μg/mL; tetracycline, 10 μg/mL; and apramycin, 50 μg/mL. The N-minimal medium (5 mM KCl, 7.5 mM (NH_4_)_2_SO_4_, 0.5 mM K_2_SO_4_, 1 mM KH_2_PO_4_, 0.1 M Tris, and 0.1% casamino acids) was supplemented with glycerol and MgCl_2_ as indicated (10, 61). The pH was adjusted as indicated.

### β-galactosidase assays *in vitro* and in mice.

β-galactosidase assays were performed using a microtiter plate assay as previously described (62). Bacteria were grown in LB medium for 16 h, subcultured 1/100 in 3 mL of N-minimal medium (pH 5.6) containing 38 mM glycerol and 10 mM or 10 μM MgCl_2_, and then grown for 3 h at 37°C on a roller drum in 12 × 75 mm Falcon tubes. The β-galactosidase activity units are defined as (μmol of ortho-nitrophenol formed per minute) × 10^6^/(OD_600_ × mL of cell suspension) and are presented as the mean ± standard deviation with *n* = 3.

BALB/c mice were infected intraperitoneally with 10^4^ cells of the in-locus *paeA-lac* fusion strain that were grown in LB medium for 16 h. After 4 days of infection, the mice were sacrificed, and their spleens were homogenized. Bacterial cells were extracted from the splenic tissue using a protocol based on (63). The spleens were homogenized in 1 mL of sterile phosphate buffered saline (PBS) and centrifuged for 2 min. The supernatants were discarded. The pellets were resuspended in 1 mL of sterile dH_2_O. 40 U of DNase I were added and incubated for 20 min at room temperature (RT) with periodic mixing. The samples were centrifuged for 2 min, and the supernatants were discarded. The pellets were washed twice with sterile Z-buffer (64) and were resuspended in 500 μL of sterile Z-buffer. 20 μL aliquots were removed from each sample, and serial dilutions in PBS were plated on LB agar for CFU determination. Then, β-mercaptoethanol was added to each sample to a final concentration of 40 mM, followed by 1 drop of 0.1% SDS and 2 drops of chloroform. The samples were then vortexed for 15 to 20 sec and left at RT for 10 to 15 min in order to allow the chloroform to settle. The β-galactosidase activity in the resulting samples was assayed using the chemiluminescent substrate Lumigal 530 (30 min incubation at 37°C; 5 sec read in a Berthold, Inc. Lumat LB9501 luminometer), according to the manufacturer's instructions (Lumigen, Inc.). The β-galactosidase activity of each sample was calculated per CFU of bacteria in the sample. The β-galactosidase activity in bacteria grown *in vitro* in LB medium (16 h of growth) was measured using the same assay and was calculated per CFU of bacteria in the sample.

### Survival assay.

Overnight cultures, grown in N-minimal medium (pH 7.4) supplemented with 15 mM glycerol and 10 mM MgCl_2_, were diluted 200-fold into the same medium and grown for 4 h. The cells were then washed with 0.85% NaCl three times, diluted to an OD_600_ value of either 0.005 or 0.05 into 10 mL of N-minimal medium (pH 7.4 or 5.5) with 15 mM glycerol, with or without 10 mM MgCl_2_, in a 125 mL baffled flask, and incubated at 37°C. Before and after incubation, serial dilutions of the cultures were plated on LB agar plates supplemented with 0.1% glucose and were incubated overnight at 37°C to determine CFU. For the growth curves, the OD_600_ of 250 μL of culture was measured in a BioTek ELx808 Absorbance Reader at the indicated time points. When necessary, the cultures were diluted in the original growth medium to accurately measure the OD_600_ value.

### Polyamine measurements.

To collect enough cells at the early stages of low Mg^2+^ stress, we started cultures using a 10-fold higher cell density than those used in the experiments described above. First, we confirmed that the increased inoculum did not affect the phenotypes of the various mutant strains under Mg^2+^ starvation conditions (Fig. S3). To measure the intracellular polyamine levels, cells were collected via centrifugation (7,000 × *g*) at the indicated time points and washed twice with 0.85% NaCl. The supernatant was removed. The cell pellet was frozen at −80°C and then suspended in 1 mL of methanol and disrupted via sonication. The samples were cleared via centrifugation, and the concentration of polyamines in the solvent was determined via liquid chromatography-mass spectrometry (LC-MS) at the Metabolomics Center, Roy J. Carver Biotechnology Center, University of Illinois at Urbana-Champaign. The concentration is reported as nmoles per (mL × OD_600_) and is converted to an intracellular concentration assuming 7.97 × 10^8^ CFU per 1 ml of OD_600_ = 1 culture (empirically determined) and 2.3 × 10^−15^ L per cell (65).

10.1128/mbio.02698-22.3 FIG S3 The survival phenotypes of various mutants under Mg^2+^ starvation conditions are not affected by an increased inoculum. The indicated strains were pre-grown to the mid-exponential phase in N-minimal medium with 10 mM MgCl_2_, washed, and diluted into N-minimal medium with or without 10 mM MgCl_2_ at a starting OD_600_ of 0.05 (instead of an OD_600_ of 0.005, as in Fig. 2). Cultures were incubated at 37°C, and CFUs were measured at the indicated time points. CFUs are presented as the mean ± SD, *n* = 6. Unpaired *t* test (*, *P* < 0.05; **, *P* < 0.005; ***, *P* < 0.0005; black labels) versus the *paeA*^+^ wild-type background + Mg^2+^ or (*, *P* < 0.05; **, *P* < 0.005; ***, *P* < 0.0005; gray labels) versus the *paeA*^+^ wild-type background minus Mg^2+^. Strains used: 14028, JS2430, JS2560, JS2561, JS2562, JS2598, JS2563, and JS2600. Download FIG S3, TIF file, 2.1 MB. Copyright © 2022 Iwadate et al. 2022 Iwadate et al. https://creativecommons.org/licenses/by/4.0/ This content is distributed under the terms of the Creative Commons Attribution 4.0 International license.

### Mg^2+^ measurements via inductively coupled plasma-mass spectrometry (ICP-MS).

The cells were cultured as described above for the survival assays, except that the total volume of each culture was 80 mL grown in a 1 L baffled flask. After 0, 3, and 9 h, cells were collected by centrifugation (7,000 × *g*) and washed twice with 0.85% NaCl. The cell pellets were dried at room temperature and were sent to the University of Georgia Center for Applied Isotope Studies for the determination of the total Mg^2+^ content. The concentration is reported as μg per g of dry weight and is converted to an intracellular concentration assuming 2.8 × 10^−13^ g per cell (66) and 2.3 × 10^−15^ L per cell (65).

### Polyamine sensitivity assay.

Polyamine stocks were prepared at 50 mM in 0.85% NaCl with HEPES buffer (pH 8.5). Cultures that were grown overnight in N-minimal medium (pH 7.4) supplemented with 15 mM glycerol and 10 mM MgCl_2_ were diluted into the same medium and grown for 4 h. The cells were then washed with 0.85% NaCl three times, diluted to an OD_600_ value of 0.05 into N-minimal medium (pH 7.4) with the indicated amount of MgCl_2_, and incubated for 4 h at 37°C. Then, the cells were washed three times, diluted to an OD_600_ value of 0.05 in 1 mL of 0.85% NaCl with HEPES buffer (pH 8.5) with the indicated amount of cadaverine, putrescine, or spermidine, and incubated at 37°C in 13 × 100 mm test tubes. At 0 and 24 h, serial dilutions of the cultures were plated on LB agar plates supplemented with 0.1% glucose, and they were incubated overnight at 37°C to determine the CFU.

### Animal assays.

All of the animal work was reviewed and approved by the University of Illinois IACUC and was performed under the protocol number 21197. The competition experiments were performed using 5 to 6-week-old mice. The BALB/cAnNHsd and C3H/HeNHsd mice were purchased from Envigo. The Salmonella strains were grown overnight in LB medium, mixed 1:1, and diluted to a target inoculum of approximately 1,000 CFU in 200 μL sterile PBS. For the Δsynth background strains, we used an inoculum of approximately 10,000 CFU. The mice were infected via the intraperitoneal route. Each inoculum was plated on LB medium to measure the total inoculum and was replica plated to the appropriate selective medium in order to calculate the input ratio for each strain. After 4 days of infection for the BALB/cAnNHsd mice or 5 days of infection for the C3H/HeNHsd mice, the animals were sacrificed via CO_2_ asphyxiation, and their spleens were removed and homogenized. Serial dilutions of the spleen homogenates were plated on LB medium and were replica plated to the appropriate selective medium in order to calculate the output ratio for each competition. The competitive index (CI) was calculated as (percent strain A recovered/percent strain B recovered)/(percent strain A inoculated/percent strain B inoculated). The statistical comparisons of individual competitions were done using Student’s *t* tests.
