# Supplementary figures and images for: Cation Homeostasis: Coordinate Regulation of Polyamine and Magnesium Levels in Salmonella
Source: mBio. 2022 Dec 7;14(1):e02698-22. doi: 10.1128/mbio.02698-22 (PMC9972920; doi:10.1128/mbio.02698-22)

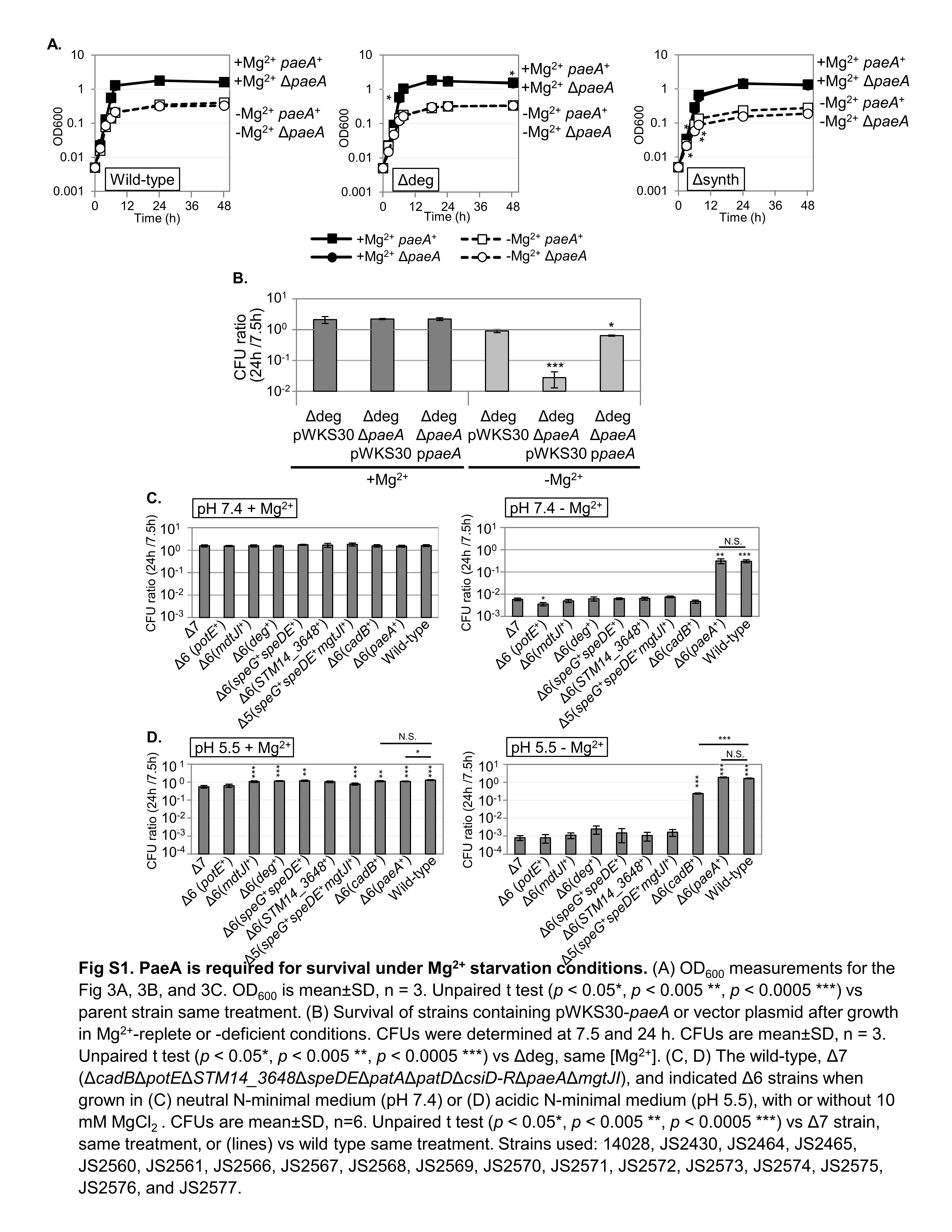

Supplement: FIG S1 [file mbio.02698-22-s0005.tif]

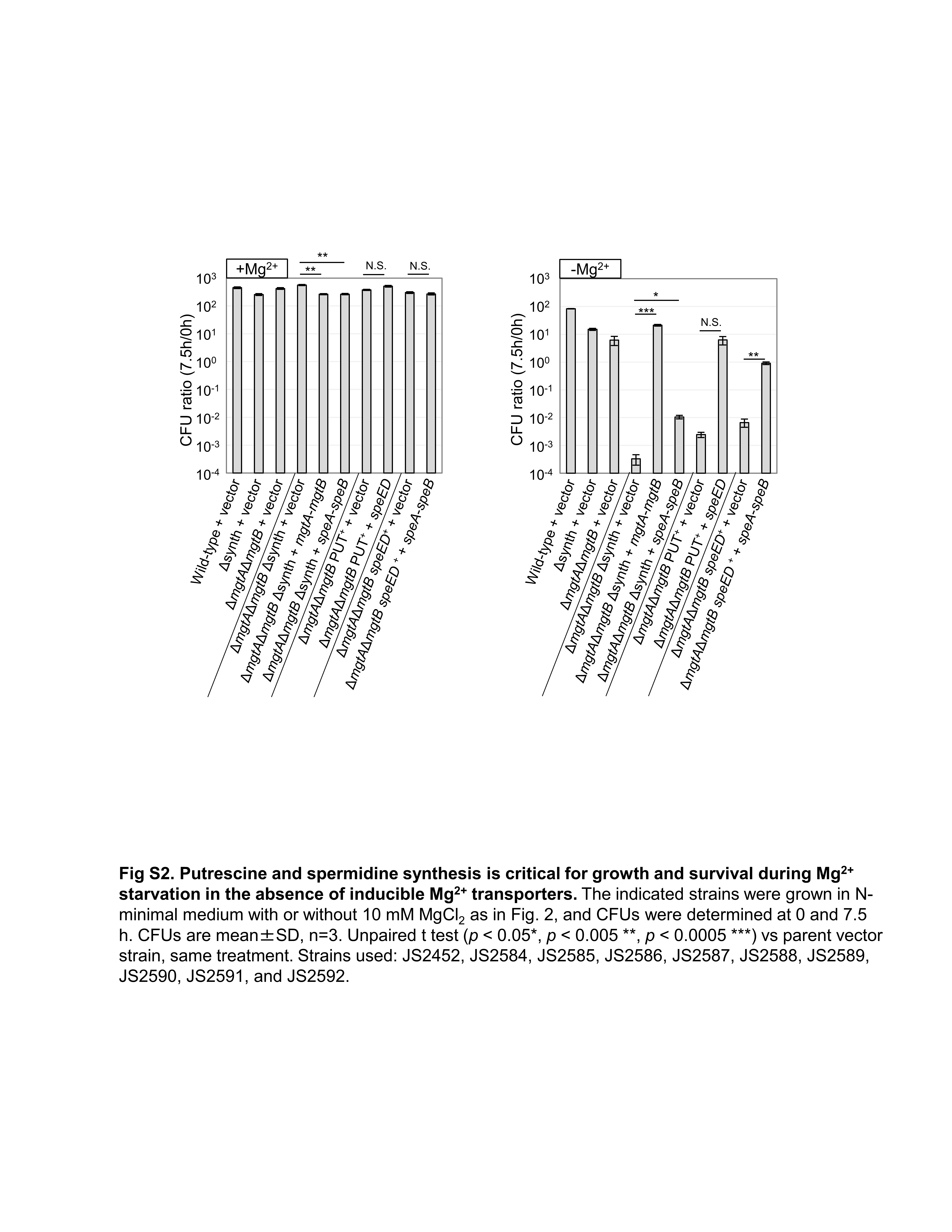

Supplement: FIG S2 [file mbio.02698-22-s0006.tif]

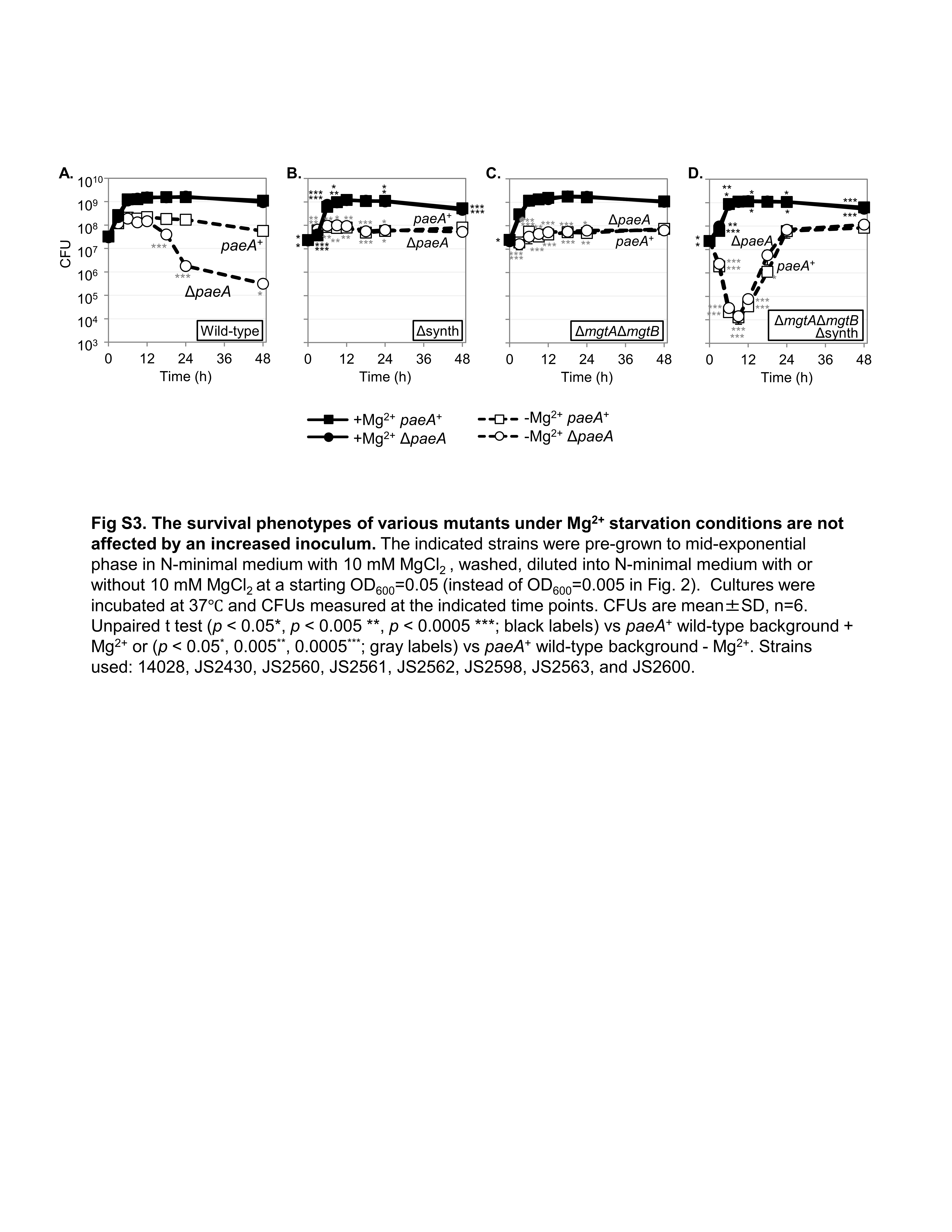

Supplement: FIG S3 [file mbio.02698-22-s0007.tif]
