# Supplementary material for: Cation Homeostasis: Coordinate Regulation of Polyamine and Magnesium Levels in Salmonella
Source: mBio. 2022 Dec 7;14(1):e02698-22. doi: 10.1128/mbio.02698-22 (PMC9972920; doi:10.1128/mbio.02698-22)
Supplement: TABLE S1 [file mbio.02698-22-s0002.docx]

Table S1. Strain list

| **Strain**^a^ | **Genotype** | **Deletion endpoint**^b^ | **Source or reference**^c^ |
| --- | --- | --- | --- |
| DH5αλpir^+^ | E. coli endA1 hsdR17 supE44 thi-1 recA1 gyrA relA1 DEL(lac-argF)U169 deoR φ80 Δ(lac)M15 λ pir^+^ |  | Lab stock |
| 14028 | *Salmonella* Typhimurium |  | Lab stock (ATCC)^d^ |
| JS198 | LT2 metE551 metA22 ilv452 trpB2 hisC527(am) galE496 xyl-404 rpsL120 flaA66 hsdL6 hsdSA29 zjg8103::pir^+^ recA1 |  | (1) |
| JS2430 | Δ*paeA*::*Cat* | *paeA*: 4656506 - 4657849 | (2) |
| JS2452 | 14028 pWKS30 |  | (2) |
| JS2464 | Δ*patA* Δ*patD* Δ*csiD-R* | *patA*: 3402931 - 3404339, *patD*: 1696811 - 1698235, *csiD-R*: 2958076 - 2965364 | (2) |
| JS2465 | Δ*patA* Δ*patD* Δ*csiD-R* Δ*paeA*::*Cat* |  | (2) |
| JS2560 | Δ*speED* Δ*speG* Δ*speF* Δ*cadA* Δ*speC* Δ*ldcC* Δ*speB* Δ*speA*::*Tc* | *speED*: 194906 - 196581, *speG*: 1590727 - 1591248  *speF*: 766074 - 763920 *cadA*: 2751503 - 2753647 *speC*: 3291026 - 3293161 *ldcC*: 276726 - 278867 *speB*: 3258658 - 3259578 *speA*: 3268047 - 3270024 |  |
| JS2561 | Δ*speED* Δ*speG* Δ*speF* Δ*cadA* Δ*speC* Δ*ldcC* Δ*speB* Δ*speA*::*Tc* Δ*paeA*::*Cat* |  |  |
| JS2562 | Δ*mgtA*::*Kan* Δ*mgtB* | *mgtA*: 4712521 - 4715229  *mgtB*: 3975237 - 3977957 |  |
| JS2563 | Δ*speED* Δ*speG* Δ*speF* Δ*cadA* Δ*speC* Δ*ldcC* Δ*speB* Δ*speA*::*Tc* Δ*mgtA*::*Kan* Δ*mgtB* |  |  |
| JS2564 | Δ*patA* Δ*patD* Δ*csiD-R* Δ*mgtA* Δ*mgtB* |  |  |
| JS2565 | Δ*patA* Δ*patD* Δ*csiD-R* Δ*mgtA* Δ*mgtB* Δ*paeA*::*Cat* |  |  |
| JS2566 | Δ*patA* Δ*patD* Δ*csiD-R* pWKS30 empty |  |  |
| JS2567 | Δ*patA* Δ*patD* Δ*csiD-R* Δ*paeA*::*Cat* pWKS30 empty |  |  |
| JS2568 | Δ*patA* Δ*patD* Δ*csiD-R* Δ*paeA*::*Cat* pWKS30-P*_lacZ_paeA* |  |  |

| JS2569 | Δ*patA* Δ*patD* Δ*csiD-R* Δ*cadB* Δ*potE* Δ*paeA* Δ*STM14_3648* Δ*speG* Δ*speED* Δ*mgtJI*::*Kan* | *cadB*: 2750089 - 2751420, *potE*: 762560 - 763879, *STM14-3648*: 3201717 – 3202187  *STM14-3648*: 3201717 - 3202187  *mgtJI*: 1570231 - 1570903 |  |
| --- | --- | --- | --- |
| JS2570 | Δ*patA* Δ*patD* Δ*csiD-R* Δ*cadB* Δ*paeA* Δ*STM14_3648* Δ*speG* Δ*speED* Δ*mgtJI*::*Kan* |  |  |
| JS2571 | Δ*patA* Δ*patD* Δ*csiD-R* Δ*cadB* Δ*potE* Δ*paeA* Δ*STM14_3648* Δ*speG* Δ*speED* |  |  |
| JS2572 | Δ*cadB* Δ*potE* Δ*paeA* Δ*STM14_3648* Δ*speG* Δ*speED* Δ*mgtJI*::*Kan* |  |  |
| JS2573 | Δ*patA* Δ*patD* Δ*csiD-R* Δ*cadB* Δ*potE* Δ*paeA* Δ*STM14_3648* Δ*mgtJI*::*Kan* |  |  |
| JS2574 | Δ*patA* Δ*patD* Δ*csiD-R* Δ*cadB* Δ*potE* Δ*paeA* Δ*speG* Δ*speED* Δ*mgtJI*::*Kan* |  |  |
| JS2575 | Δ*patA* Δ*patD* Δ*csiD-R* Δ*cadB* Δ*potE* Δ*paeA* Δ*STM14_3648* |  |  |
| JS2576 | Δ*patA* Δ*patD* Δ*csiD-R* Δ*potE* Δ*paeA* Δ*STM14_3648* Δ*speG* Δ*speED* Δ*mgtJI*::*Kan* |  |  |
| JS2577 | Δ*patA* Δ*patD* Δ*csiD-R* Δ*cadB* Δ*potE* Δ*STM14_3648* Δ*speG* Δ*speED* Δ*mgtJI*::*Kan* |  |  |
| JS2578 | Δ*speED* Δ*speG* Δ*speF* Δ*speC* Δ*speB* Δ*speA*::*Tc* Δ*mgtA*::*Kan* Δ*mgtB* |  |  |
| JS2579 | Δ*speED* Δ*speG* Δ*cadA* Δ*ldcC* Δ*mgtA*::*Kan* Δ*mgtB* |  |  |
| JS2580 | Δ*speED* Δ*speG* Δ*speF* Δ*cadA* Δ*speC* Δ*ldcC* Δ*mgtA*::*Kan* Δ*mgtB* |  |  |
| JS2581 | Δ*speED* Δ*speG* Δ*cadA* Δ*ldcC* Δ*speB* Δ*speA*::*Tc* Δ*mgtA*::*Kan* Δ*mgtB* |  |  |
| JS2582 | Δ*speG* Δ*cadA* Δ*ldcC* Δ*mgtA*::*Kan* Δ*mgtB* *speED* + *Apr* |  |  |
| JS2583 | Δ*speG* Δ*speF* Δ*cadA* Δ*speC* Δ*ldcC* Δ*mgtA*::*Kan* Δ*mgtB* *speED* + *Apr* |  |  |
| JS2584 | Δ*speED* Δ*speG* Δ*speF* Δ*cadA* Δ*speC* Δ*ldcC* Δ*speB* Δ*speA*::*Tc* + pWKS30 empty |  |  |
| JS2585 | Δ*mgtA*::*Kan* Δ*mgtB* + pWKS30 empty |  |  |
| JS2586 | Δ*speED* Δ*speG* Δ*speF* Δ*cadA* Δ*speC* Δ*ldcC* Δ*speB* Δ*speA*::*Tc* Δ*mgtA*::*Kan* Δ*mgtB* + pWKS30 empty |  |  |
| JS2587 | Δ*speED* Δ*speG* Δ*speF* Δ*cadA* Δ*speC* Δ*ldcC* Δ*speB* Δ*speA*::*Tc* Δ*mgtA*::*Kan* Δ*mgtB* + pWKS30-*mgtA*-*mgtB* |  |  |
| JS2588 | Δ*speED* Δ*speG* Δ*speF* Δ*cadA* Δ*speC* Δ*ldcC* Δ*speB* Δ*speA*::*Tc* Δ*mgtA*::*Kan* Δ*mgtB* + pWKS30-*speA*-*speB* |  |  |
| JS2589 | Δ*speED* Δ*speG* Δ*cadA* Δ*ldcC* Δ*mgtA*::*Kan* Δ*mgtB* + pWKS30 empty |  |  |
| JS2590 | Δ*speED* Δ*speG* Δ*cadA* Δ*ldcC* Δ*mgtA*::*Kan* Δ*mgtB* + pWKS30-*speED* |  |  |
| JS2591 | Δ*speG* Δ*speF* Δ*cadA* Δ*speC* Δ*ldcC* Δ*speB* Δ*speA*::*Tc speED* + *Apr* Δ*mgtA*::*Kan* Δ*mgtB* + pWKS30 empty |  |  |
| JS2592 | Δ*speG* Δ*speF* Δ*cadA* Δ*speC* Δ*ldcC* Δ*speB* Δ*speA*::*Tc* *speED* + *Apr* Δ*mgtA*::*Kan* Δ*mgtB* + pWKS30-*speA*-*speB* |  |  |
| JS2593 | Δ*speED* Δ*speG* Δ*cadA* Δ*ldcC zjd*::*Kan* |  |  |
| JS2594 | Δ*speED* Δ*speG* Δ*speF* Δ*speC* Δ*speB* Δ*speA*::*Tc* *zjd*::*Kan* |  |  |
| JS2595 | Δ*speG* Δ*cadA* Δ*ldcC* *speED* + *Apr zjd*::*Kan* |  |  |
| JS2596 | Δ*mgtB* *zjd*::*Kan* |  |  |
| JS2597 | Δ*speED* Δ*speG* Δ*speF* Δ*cadA* Δ*speC* Δ*ldcC* Δ*speB* Δ*speA*::*Tc* Δ*mgtB* *zjd*::*Kan* |  |  |
| JS2598 | Δ*mgtA*::*Kan* Δ*mgtB* Δ*paeA*::*Cat* |  |  |
| JS2599 | Δ*mgtC* | *mgtC*: 3978180 - 3979974 |  |
| JS2600 | Δ*speED* Δ*speG* Δ*speF* Δ*cadA* Δ*speC* Δ*ldcC* Δ*speB* Δ*speA*::*Tc* Δ*mgtA*::*Kan* Δ*mgtB* Δ*paeA*::*Cat* |  |  |
| JS1068 | Δ*phoPQ*::*Cat* |  | (3) |
| JS1054 | *phoQ24* Δ*ycfD612::Kan* |  | (3) |
| JS2601 | Φ(*paeA-lacZ+*) |  |  |
| JS2602 | Φ(*paeA-lacZ+*) Δ*phoPQ*::*Cat* |  |  |
| JS2603 | Φ(*paeA-lacZ+*) *phoQ24* Δ*ycfD612::Kan* |  |  |
| JS2604 | *attλ*::pDX1::P*msrA-lacZ+* |  |  |
| JS2605 | *attλ*::pDX1::P*msrA-lacZ+* Δ*phoPQ*::*Cat* |  |  |
| JS2606 | *attλ*::pDX1::P*msrA-lacZ+ phoQ24* Δ*ycfD612::Kan* |  |  |
| JS2607 | *attλ*::pDX1::P*paeA-lacZ+* |  |  |
| JS2608 | *attλ*::pDX1::P*paeA-lacZ+* Δ*phoPQ::Cm* |  |  |
| JS2609 | *attλ*::pDX1::P*paeA-lacZ+ phoQ24* Δ*ycfD612::Kan* |  |  |
| JS2610 | *attλ::*pDX1::P*msrA* *msrA*+ P*paeA-lacZ+* |  |  |
| JS2611 | *attλ::*pDX1::P*msrA* *msrA*+ P*paeA-lacZ+* Δ*phoPQ*::*Cat* |  |  |
| JS2612 | *attλ::*pDX1::P*msrA* *msrA*+ P*paeA-lacZ+ phoQ24* Δ*ycfD612::Kan* |  |  |

^a^ All *Salmonella* strains are isogenic derivatives of *S. enterica* serovar Typhimurium strain 14028.

^b^ Numbers indicate the base pairs that are deleted (inclusive) as defined in the *S. enterica* serovar Typhimurium 14028 genome sequence (National Center for Biotechnology Information; NC_016856.1)

^c^ This study, unless otherwise indicated

^d^ ATCC, American Type Culture Collection

References

1. Ellermeier CD, Janakiraman A, Slauch JM. 2002. Construction of targeted single copy *lac* fusions using lambda Red and FLP-mediated site-specific recombination in bacteria. Gene 290:153-161. <https://doi.org/10.1016/S0378-1119(02)00551-6>

2. Iwadate Y, Ramezanifard R, Golubeva YA, Fenlon LA, Slauch JM. 2021. PaeA (YtfL) protects from cadaverine and putrescine stress in *Salmonella* Typhimurium and *E. coli*. Mol Microbiol 115:1379-1394. <https://doi.org/10.1111/mmi.14686>

3. Golubeva YA, Sadik AY, Ellermeier JR, Slauch JM. 2012. Integrating global regulatory input into the *Salmonella* pathogenicity island 1 type III secretion system. Genetics 190:79-90. <https://doi.org/10.1534/genetics.111.132779>
