# Supplementary material for: Cation Homeostasis: Coordinate Regulation of Polyamine and Magnesium Levels in Salmonella
Source: mBio. 2022 Dec 7;14(1):e02698-22. doi: 10.1128/mbio.02698-22 (PMC9972920; doi:10.1128/mbio.02698-22)
Supplement: TABLE S2 [file mbio.02698-22-s0003.docx]

Table S2. Plasmid list

| **Name** | **Characteristic** | **Cloned endpoint**^a^ | **Source or reference**^b^ |
| --- | --- | --- | --- |
| pKD46 | bla PBAD gam bet exo pSC101 oriTS |  | (1) |
| pCP20 | bla cat cI857 λPR flp pSC101 oriTS |  | (1) |
| pKD3 | bla FRT cat FRT PS1 PS2 oriR6K |  | (1) |
| pKD4 | bla FRT aph FRT PS1 PS2 oriR6K |  | (1) |
| pKD13 | bla FRT ahp FRT PS1 PS4 oriR6K |  | (1) |
| pWKS30 | bla pSC101 ori |  | (2) |
| pWKS30-P*_lacZ_paeA* |  | 4656491 - 4657863 | (3) |
| pWKS30-*mgtA*-*mgtB* |  | *mgtA*: 4712138 - 4715362 *mgtB*: 3975229 - 3979334 |  |
| pWKS30-*speA*-*speB* |  | *speA*: 3267950 - 3270141 *speB*: 3258524 - 3259741 |  |
| pWKS30-*speED* |  | 194910 - 197201 |  |
| pDX1 | *lacZ* t_L3_ λ*attP* oriR6K *aacIV* t_mgB_ |  | (4) |
| pDX1-P*msrA* |  | 4658671-4658892 |  |
| pDX1-P*paeA* |  | 4657914-4658026 |  |
| pDX1-P*msrA* P*paeA* |  | 4657914-4658892 |  |

^a^ Numbers indicate the base pairs that are cloned (inclusive) as defined in the *S*. enterica serovar Typhimurium 14028 genome sequence (National Center for Biotechnology Information; NC_016856.1)

^b^ This study, unless otherwise indicated

References

1. Datsenko KA, Wanner BL. 2000. One-step inactivation of chromosomal genes in *Escherichia coli* K-12 using PCR products. Proc Natl Acad Sci USA 97:6640-6645. <https://doi.org/10.1073/pnas.120163297>

2. Wang RF, Kushner SR. 1991. Construction of versatile low-copy-number vectors for cloning, sequencing and gene expression in *Escherichia coli*. Gene 100:195-199.

3. Iwadate Y, Ramezanifard R, Golubeva YA, Fenlon LA, Slauch JM. 2021. PaeA (YtfL) protects from cadaverine and putrescine stress in *Salmonella* Typhimurium and *E. coli*. Mol Microbiol 115:1379-1394. <https://doi.org/10.1111/mmi.14686>

4. Lin D, Rao CV, Slauch JM. 2008. The *Salmonella* SPI1 type three secretion system responds to periplasmic disulfide bond status via the flagellar apparatus and the RcsCDB system. J Bacteriol 190:87-97. <https://doi.org/10.1128/JB.01323-07>
