# Supplementary material for: Cation Homeostasis: Coordinate Regulation of Polyamine and Magnesium Levels in Salmonella
Source: mBio. 2022 Dec 7;14(1):e02698-22. doi: 10.1128/mbio.02698-22 (PMC9972920; doi:10.1128/mbio.02698-22)
Supplement: TEXT S1 [file mbio.02698-22-s0001.docx]

**Plasmid construction**

Plasmids are listed in Table S2. All plasmids were passaged through a restriction-minus modification-plus Pi^+^ *Salmonella* strain JS198 prior to transformation into *Salmonella* strains. All plasmid constructs were confirmed by DNA sequencing.

Primers are listed in Table S3 and were purchased from IDT, Inc. To construct the pWKS30- *mgtA* *mgtB* plasmid, we first made pWKS30-*mgtA*. The *mgtA* fragment (amplified using primers YIS_471-2 and YIS_471-4) and pWKS30 fragment (primers YIV_pWKS30-12 and YIV_pWKS30-19) were combined by PCR using primers YIS_471-3 and YIV_pWKS30-11. The resulting fragment was phosphorylated by T4 Polynucleotide Kinase (New England Biolabs), ligated by T4 DNA Ligase (New England Biolabs) according to the manufacturer's protocols, and transformed into *E. coli* strain DH5αλpir^+^. Then, Δ*mgtC* strain (JS2599) was used as a template to amplify *mgtB* gene using primers YIS_397-30 and YIS_397-31. The resulting *mgtB* fragment and pWKS30-*mgtA* fragment (amplified from pWKS30- *mgtA* using primers YIV_pWKS30-15 and YIV_pWKS30-21) were combined using NEBuilder® HiFi DNA Assembly (New England Biolabs) according to the manufacturer's protocol, and transformed into *E. coli* strain DH5αλpir^+^.

To construct the pWKS30- *speA* -*speB* plasmid, we first made pWKS30- *speA*. The *speA* fragment (amplified by primers YIS_326-1 and YIS_327-1) and pWKS30 fragment (primers YIV_pWKS30-12 and YIV_pWKS30-19) were fused by overlap PCR using primers YIS_326-8 and YIV_pWKS30-11. The resulting fragment was phosphorylated, ligated, and transformed into *E. coli* strain DH5αλpir^+^. The *speB* fragment (primers YIS_325-1 and YIS_326-3) and pWKS30-*speA* plasmid fragment (amplified from the pWKS30-*speA* template using primers YIV_pWKS30-14 and YIV_pWKS30-6) were combined by NEBuilder® HiFi DNA Assembly (New England Biolabs) according to the manufacturer's protocol, and transformed into *E. coli* strain DH5αλpir^+^.

To construct pWKS30-*speED*, the *speED* fragment (amplified using primers YIS_19-1 and YIS_19-6) and pWKS30 fragment (primers YIV_pWKS30-5 and YIV_pWKS30-15) were fused by overlap PCR using primers YIS_19-9 and YIV_pWKS30-21. The resulting fragment was phosphorylated, ligated using, and transformed into *E. coli* strain DH5αλpir^+^ as described for the pWKS30-*mgtA* plasmid
