# Supplementary material for: Cation Homeostasis: Coordinate Regulation of Polyamine and Magnesium Levels in Salmonella
Source: mBio. 2022 Dec 7;14(1):e02698-22. doi: 10.1128/mbio.02698-22 (PMC9972920; doi:10.1128/mbio.02698-22)
Supplement: TABLE S3 [file mbio.02698-22-s0004.docx]

Table S3. Primer list

| **Name** | **Sequence (5' to 3)** | **Usage** |
| --- | --- | --- |
| YIS_471-2 | CTACAGTTGCTTGAATAAGCCC | pWKS30-*mgtA*-*mgtB* |
| YIS_471-3 | TCGCGTTAAACTTGTCGAGC | pWKS30-*mgtA*-*mgtB* |
| YIS_471-4 | GTTTCCCGACTGGAAAGCGGGCAGTG  AGCGCAACGCAATCCTGTTTTCCCGTGGCGTGA | pWKS30-*mgtA*-*mgtB* |
| YIV_pWKS30-11 | /5Phos/AACGTCGTGACTGGGAAAACC | pWKS30-*mgtA*-*mgtB* and pWKS30-*speA*-*speB* |
| YIV_pWKS30-12 | ATTGCGTTGCGCTCACTGCC | pWKS30-*mgtA*-*mgtB* and pWKS30-*speA*-*speB* |
| YIV_pWKS30-19 | CGATAAGCTTGATATCGAATTCCTGC | pWKS30-*mgtA*-*mgtB* and pWKS30-*speA*-*speB* |
| YIS_397-30 | GCATTCGCCATTCAGGCTGCGAGCTAT  CGCCGGTATTAAGC | pWKS30-*mgtA*-*mgtB* |
| YIS_397-31 | GAGATCTCTACGGGTCGGATCATTATT  AAAACCACTGGCC | pWKS30-*mgtA*-*mgtB* |
| YIV_pWKS30-15 | GCAGCCTGAATGGCGAATGC | pWKS30-*mgtA*-*mgtB* |
| YIV_pWKS30-21 | /5Phos/ATCCGACCCGTAGAGATCTC | pWKS30-*mgtA*-*mgtB* |
| YIS_326-1 | GTGCTGATTCCCATAACGCA | pWKS30-*speA*-*speB* |
| YIS_327-1 | GTTTCCCGACTGGAAAGCGGGCAGTGAG  CGCAACGCAATAAAAAGAAACCGGTCGCGCAG | pWKS30-*speA*-*speB* |
| YIS_326-8 | GGGATTGTAGGCCCATAAGA | pWKS30-*speA*-*speB* |
| YIS_325-1 | GGTTGCCAGAATATGGACTC | pWKS30-*speA*-*speB* |
| YIS_326-3 | TTGATTCACGGTGGTGGTCT | pWKS30-*speA*-*speB* |
| YI_pWKS30-6 | GAATCAGGGGATAACGCAGG | pWKS30-*speA*-*speB* |
| YIV_pWKS30-14 | TGTGGATAACCGTATTACCG | pWKS30-*speA*-*speB* |
| YIS_19-1 | GTCGTCGATTACCAGCGTAAAG | pWKS30-*speED* |
| YIS_19-6 | GAAGGCTCTCAAGGGCATCGGCATTCGCCAT  TCAGGCTGCTGAACAGGCGGGTGTCTAAG | pWKS30-*speED* |
| YIV_pWKS30-5 | CTCCTACCAAGACGACTTCA | pWKS30-*speED* |
| YIV_pWKS30-15 | GCAGCCTGAATGGCGAATGC | pWKS30-*speED* |
| YIS_19-9 | GCTGCCAGTGGTGAATCAGA | pWKS30-*speED* |
| YIV_pWKS30-21 | /5Phos/ATCCGACCCGTAGAGATCTC | pWKS30-*speED* |
| *msrA* PF | CGGTACCTATGTGGCACATTTTCTCCT | for cloning into pDX1 |
| *msrA* ATGR | CGAATTCGAGCTATTCTCCCGAAAGCGT | for cloning into pDX1 |
| *paeA* SPF | CGGTACCTGGCGTAGGGGGAACTTGT | for cloning into pDX1 |
| *paeA* D2051R2 | CGAATTCATGAAAACCGGCTCGTAGCA | for cloning into pDX1 |
| pDX1 CF | AGGACGCCCGCCATAAACT |  |
| pDX1 CR | GCCAGTGAATCCGTAATCATG |  |
